# Microwave-Hydrothermal Tuning of Spinel-Type Co_*3*_O_*4*_ Water Oxidation Catalysts

**DOI:** 10.3389/fchem.2020.00473

**Published:** 2020-06-09

**Authors:** Karla Lienau, C. A. Triana, Lukas Reith, Sebastian Siol, Greta R. Patzke

**Affiliations:** ^1^Department of Chemistry, University of Zurich, Zurich, Switzerland; ^2^Empa—Swiss Federal Laboratories for Materials Science and Technology, Dübendorf, Switzerland

**Keywords:** Co_3_O_4_ spinel, microwave-hydrothermal synthesis, water oxidation, electrocatalysis, synthesis parameters

## Abstract

Water oxidation is the bottleneck reaction for overall water splitting as a direct and promising strategy toward clean fuels. However, the development of robust and affordable heterogeneous water oxidation catalysts remains challenging, especially with respect to the wide parameter space of synthesis and resulting material properties. Oxide catalysts performance in particular has been shown to depend on both synthetic routes and applied catalytic test methods. We here focus on spinel-type Co_3_O_4_ as a representative case for an in-depth study of the influence of rather subtle synthetic parameter variations on the catalytic performance. To this end, a series of Co_3_O_4_ samples was prepared via time-saving and tunable microwave-hydrothermal synthesis, while systematically varying a single parameter at a time. The resulting spinel-type catalysts were characterized with respect to key materials properties, including crystallinity, oxidation state and surface area using a wide range of analytical methods, such as PXRD, Raman/IR, XAS and XPS spectroscopy. Their water oxidation activity in electrocatalytic and chemical oxidation setups was then compared and correlated with the obtained catalyst properties. Both water oxidation methods displayed related trends concerning favorable synthetic parameters, namely higher activity for lower synthesis temperatures, lower precursor concentrations, addition of hydrogen peroxide and shorter ramping and reaction times, respectively. In addition to the surface area, structural features such as disorder were found to be influential for the water oxidation activity. The results prove that synthetic parameter screening is essential for optimal catalytic performance, given the complexity of the underlying performance-properties relationships.

## Introduction

Water splitting is a promising renewable energy approach due to the superior storage options of fuels compared to electricity generated by solar cells. After decades of research into water splitting, the optimal catalytic systems are yet to be found, not only in terms of efficiency but also considering stability and applicability (Chu et al., 2017). To this end, the water oxidation half reaction with its four electron transfer steps remains the bottleneck of the process. Heterogeneous water oxidation catalysts (WOCs) are the most obvious choice for future technical applications due to their superior stability and scale-up possibilities compared to molecular catalysts (Najafpour et al., 2016; Li et al., 2017). A widely used heterogeneous catalyst is spinel-type Co_3_O_4_ due to its several advantages, such as long time durability and lower costs compared to noble metal materials (Artero et al., 2011; Wang et al., 2016a; Zhao et al., 2017). Furthermore, Co_3_O_4_ is promising for many other applications, such as data storage, sensor applications or battery electrodes as well as for other heterogeneous catalysis processes (Wang et al., 2004, 2015; Li et al., 2005; Luo et al., 2014; Iosub and Stahl, 2015; Zhou et al., 2015; Gao et al., 2018). Heterogeneous WOCs optimization is a complex process that does not only focus on the type of catalyst, but also on other influential parameters, including crystallinity, morphology, size, exposed facets, surface vs. bulk oxidation states and other material properties (Najafpour et al., 2012; Zhao et al., 2017). In order to derive clear design guidelines for heterogeneous WOCs, the investigation of their reaction mechanisms is indispensable. However, this remains a challenging task and requires sophisticated *in situ* analyses. In a recent representative study on Co_3_O_4_ WOCs by Zhang et al. (2014a), the HO–Co_2_(μ-O/OH)_2_-OH edge site cobalt motif, i.e., two adjacent Co ions coupled via an oxygen bridge, was shown to be essential for efficient catalysis.

In search of empirical guidelines for catalyst optimization, a number of different synthesis methods have been applied in previous studies to obtain Co_3_O_4_ in various particle sizes and morphologies (Grzelczak et al., 2013; Rosen et al., 2014; Zhang et al., 2014a,b, 2017, 2018; Bergmann et al., 2015; Chua et al., 2016; Wang et al., 2016b; Cordeiro and Carvalho, 2018; Liu et al., 2018; Wei et al., 2018; Zhou et al., 2018). We recently conducted a screening investigation where we compared spinel-type Co_3_O_4_ samples prepared by a broad spectrum of different synthesis methods with respect to their characteristics and their photo-, electro-, and chemical water oxidation activity (Reith et al., 2019). Indeed we found that the applied synthetic method exerted a major influence on the catalytic water oxidation. Furthermore, considerable deviations between the activity trends for the three applied water oxidation methods were observed. Whereas a strong correlation between the activity and the surface area, crystallinity and disorder, respectively, of the corresponding materials was found for chemical oxidation, electrocatalytic water oxidation was barely influenced by these parameters. This finding agrees with the previously observed *in situ* formation of cobalt oxyhydroxide in electrocatalytic water oxidation as a general intermediate observed in previous works (Bergmann et al., 2015; Tung et al., 2015).

In the present study, we conduct an in-depth investigation of the influence of microwave-hydrothermal synthesis parameters on the WOC activity of Co_3_O_4_ nanocubes, thereby narrowing the parameter range further down in search of optimization guidelines. Microwave synthesis, which is already well-established as a versatile option in organic synthesis, attracts increasing interest in inorganic synthesis, especially for different oxide materials (Bilecka et al., 2009; Bilecka and Niederberger, 2010; Hilaire et al., 2014; Kuzmanoski et al., 2015; Dong et al., 2016; Wang et al., 2016b). Microwaves as non-ionizing and long-wavelength electromagnetic radiation enable high penetration depths and fast heating, thus rendering syntheses economically attractive by decreasing reaction times and temperatures. Due to optimization of the microwave reactor conditions, such as shape, constant motion of the reactor vessels and stirring, thermal gradients and “hot spots” can be largely prevented leading to uniform growth and dimensions of the oxide particles (Nüchter et al., 2004). This renders microwave synthesis a crucial option for well-defined particle fabrication in nanoscience and technology (Hilaire et al., 2014). However, little is still known about the growth mechanisms of metal oxides under the influence of microwaves (Moura et al., 2010; Koziej et al., 2013; Zeng et al., 2013). Therefore, we here provide a broader insight into the crucial influence of a set of different synthesis parameters.

In the case of Co_3_O_4_ we recently demonstrated that the growth mechanism can play a key role in the resulting water oxidation activity (Reith et al., 2018). Therefore, we here started from a well-defined microwave protocol for microwave-assisted spinel-type Co_3_O_4_ and systematically studied the influence of the synthetic parameters on the properties and catalytic performance of the emerging materials to demonstrate the importance of these parameters in microwave-hydrothermal WOC synthesis (Conrad et al., 2010, 2012, 2013). To this end, the Co_3_O_4_ spinel samples were characterized with a wide range of analytical methods and subsequently compared with respect to their chemical and electrocatalytic water oxidation activity. The results of this study shed detailed and practical light on our recent work by illustrating that a given Co_3_O_4_ catalyst cannot be simply compared to another specimen without taking the preparative history into account (Reith et al., 2019).

## Results and Discussions

In order to screen their influence on the WOC activity of cobalt oxide nanoparticles, the selected standard microwave hydrothermal method was varied with respect to the following parameters: temperature, precursor concentration, amount of hydrogen peroxide added to the synthesis mixture, the ramping and holding times of the synthesis and the stirring speed (see Table 1, cf. experimental section and Table 2 for details). Samples were labeled with respect to the single varied parameter.

**Table 1:** Surface area measured by N_2_-sorption and determined by BET analysis together with crystallite sizes determined from FWHM values of PXRD peaks using the Scherrer equation.

**Sample name**	**BET surface area [m^**2**^/g]**	**τ_XRD_ [nm]**
0.18 mmol	169	16
0.6 mmol	33	22
0.9 mmol	44	17
1.2 mmol	25	22
Standard	30	23
1.5 ml H_2_O_2_	67	12
3 ml H_2_O_2_	75	14
160 °C	22	18
140 °C	39	25
20 min ramping	75	14
10 min ramping	35	15
30 min holding	16	23
Fast stirring	18	32

**Table 2:** Microwave synthesis parameters of the investigated spinel-type Co_3_O_4_ samples.

**Sample name**	**Co(OAc)_**2**_ [mmol]**	**H_**2**_O_**2**_ [mL]**	**Synthesis temp [^**°**^C]**	**Ramping time [min]**	**Holding time [min]**	**Stirring speed**
0.18 mmol	**0.18**	0	180	30	45	Slow
0.6 mmol	**0.6**	0	180	30	45	Slow
0.9 mmol	**0.9**	0	180	30	45	Slow
1.2 mmol	**1.2**	0	180	30	45	Slow
**Standard**	**1.8**	**0**	**180**	**30**	**45**	**Slow**
1.5 ml H_2_O_2_	1.8	**1.5**	180	30	45	Slow
3 ml H_2_O_2_	1.8	**3**	180	30	45	Slow
160°C	1.8	0	**160**	30	45	Slow
140°C	1.8	0	**140**	30	45	Slow
20 min ramping	1.8	0	180	**20**	45	Slow
10 min ramping	1.8	0	180	**10**	45	Slow
30 min holding	1.8	0	180	30	**30**	Slow
Fast stirring	1.8	0	180	30	45	**Fast**

*Color changes indicate the variation of a single parameter (shown in bold)*.

**Figure 1:**
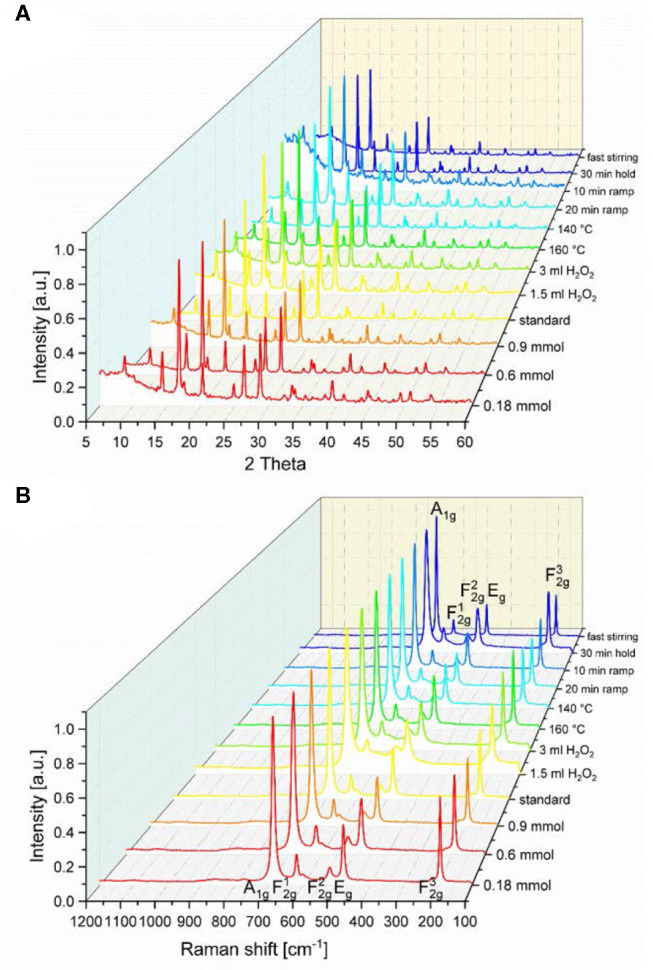
**(A)** PXRD patterns and **(B)** Raman spectra of the different spinel-type Co_3_O_4_ samples obtained from parameter variations of the standard microwave protocol.

Powder X-ray diffraction (PXRD) patterns show the formation of a phase pure spinel structure [space group *Fd*$\bar{3}$*m* (No.: 227)] for all samples (Figure 1A). Co_3_O_4_ crystallizes in the normal spinel structure, i.e., the Co^2+^ ions occupy one eighth of the tetrahedral voids of cubic close packed oxygen anions while Co^3+^ ions occupy half of the octahedral voids. The general structural model is in accordance with the obtained Raman spectra shown in Figure 1B, where five phonon excitations can be observed: the A_1g_, E_g_, and the three F_2g_ modes are Raman active (Hadjiev et al., 1988). The symmetric Co-O stretching vibration of the octahedrally coordinated cobalt centers appearing at 693–685 cm^−1^ is assigned to the A_1g_ mode (Gawali et al., 2018). The Co-O stretching vibration of tetrahedrally coordinated cobalt centers is attributed to the ${\text{F}}_{2\text{g}}^{3}$ mode at 196–193 cm^−1^. The two remaining F_2g_ modes and the E_g_ mode are located at 621–618 cm^−1^, 523–519 cm^−1^, and 484–480 cm^−1^, respectively. The relative shifts of the Raman peaks as well as the different peak broadening indicate a difference in the short-range order of the synthesized Co_3_O_4_ spinel samples, analogous to the peak broadening observed in the PXRD patterns. Only very small shifts of the Raman peak centers of A_1g_ and ${\text{F}}_{2\text{g}}^{3}$ modes are observed for the investigated samples (Figure S1), indicating the presence of very similar sample structures.

Even though the Co_3_O_4_ spinel oxides basically show the same structure, the Bragg reflections of the different samples (Figure 1A) exhibit slightly different full width half maxima (FWHM), from which the crystallite domain size was calculated by means of the Scherrer equation (Scherrer, 1918; Patterson, 1939). The so calculated crystallite sizes τ_XRD_ are compared in Table 1. Broader reflections than obtained from the standard synthesis procedure were observed for the materials synthesized with the lowest precursor concentration, with addition of H_2_O_2_ and with more rapid heating. These parameter variations resulted in a lower crystallite size for lower concentrations (τ_XRD_ ≤ 22 nm), higher amount of H_2_O_2_ (τ_XRD_ ≤ 14 nm), and shorter ramping (τ_XRD_ ≤ 15 nm), compared to the standard procedure with τ_XRD_ = 23 nm. Interestingly, the temperature, the stirring speed and the reaction time (holding time) did not exert a significant impact on the FWHM of the Bragg reflections. The values of the surface area obtained from Brunauer-Emmet-Teller (BET) analyses (Table 1) moderately correlate with the crystallite sizes obtained from Scherrer analysis, but appear generally quite low taking the crystallite sizes into account. This may indicate that the obtained Co_3_O_4_ particles were agglomerated to a large extent.

**Figure 2:**
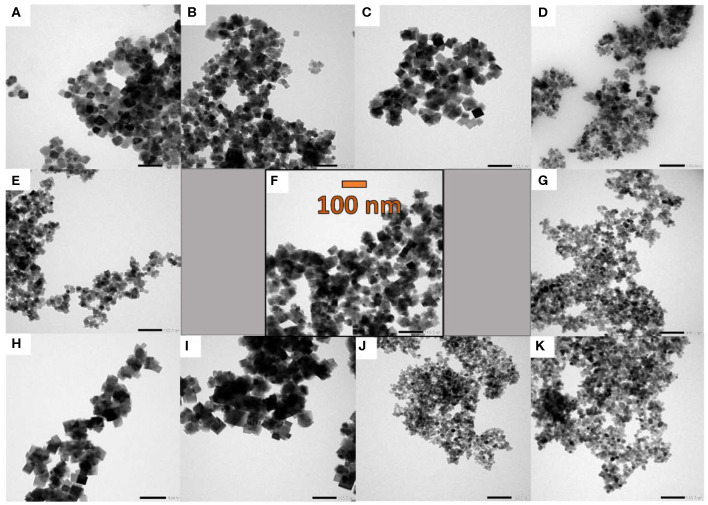
TEM images of the spinel samples emerging from parameter variations of the standard microwave protocol **(F)**: **(A)** and **(B)** synthesis at 140 and 160 °C, **(C)** 30 min holding time, **(D)** and **(G)** 10 and 20 min ramping time, **(E)** and **(H)** 0.9 and 0.18 mmol cobalt precursor, **(I)** faster stirring speed, **(J)** and **(K)** with addition of 1.5 and 3 mL H_2_O_2_ (30 wt%). For all images, the same magnification of 50 k was applied (scale bar = 100 nm).

TEM images (Figure 2) show only moderately aggregated particles, but this might be due to the different sample preparation for TEM and BET analysis. Whereas for BET measurements, the pestled powder is heated under vacuum to remove adsorbed gases, TEM samples are crushed in a mortar, followed by homogeneous dispersion in ethanol. Furthermore, it is obvious from TEM images that variation of the synthesis parameters exerts a notable influence on the resulting morphologies. Especially shorter ramping times (Figures 2D,G) and the addition of hydrogen peroxide (Figures 2J,K) result in much smaller particles with diameters around 10–20 nm, whereas particle sizes of 30–40 nm were obtained from the standard synthesis. HR-TEM and SEM images are provided in (Figures S2, S3).

**Figure 3:**
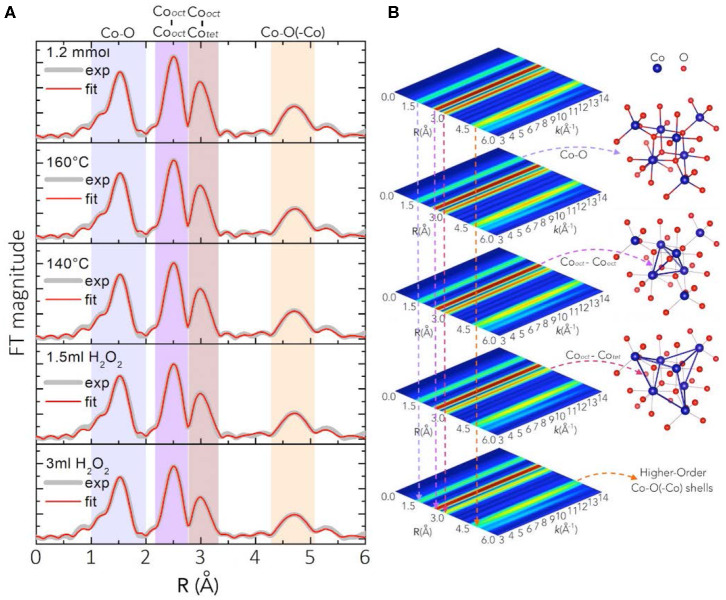
**(A)** Fitting (red) of the FT|*k*^3^χ(*k*)| spectra (gray) for Co_3_O_4_ oxides synthesized in the presence of different H_2_O_2_ amounts (3 and 1.5 mL), low temperatures of 140 and 160 °C, and a lower Co(OAc)_2_ concentration of 1.2 mmol (phase uncorrected). The 2D contour plots in **(B)** are the WT of the *k*^3^χ(*k*) spectra and their shaded regions highlight the decrease of the peak intensity due to increases in the local disorder σ^2^ of the Co-O, Co_Oct_-Co_Oct_, Co_Tet_-Co_Oct_ and higher Co-Co(-O) coordination shells.

Deeper insight into the atomic short-range order of the different synthesized Co_3_O_4_ oxides was obtained through extended X-ray absorption fine structure (EXAFS) analyses. Figure 3 shows the fitting of the Fourier-Transform FT|*k*^3^χ(*k*)| of the experimental Co *K*-edge EXAFS spectra *k*^3^χ(*k*), for oxides synthesized at different H_2_O_2_ concentrations of 3 mL and 1.5 mL, low temperatures of 140 °C and 160 °C and at a lower Co(OAc)_2_ concentration of 1.2 mmol. Calculated main values for interatomic distances, atomic coordination numbers (*N*), and Debye-Waller factors (σ^2^) are given in Table S1. The first peak in the FT|*k*^3^χ(*k*)| spectra at r ≈ 1.55 Å, arising from backscattering of neighboring O atoms, relates to Co^2+^ and Co^3+^ cations in tetrahedral {CoO_4_} and octahedral {CoO_6_} coordination with oxygen atoms at interatomic distances r ≈ 1.914 Å and r ≈ 1.899 Å, respectively. Those two shells, however, are too close to be resolved in the FT|*k*^3^χ(*k*)| spectra, and hence they convolute to a main Co-O shell with a main interatomic distance Co-O ≈ 1.913 Å and main atomic coordination number *N* = 5.333. The second and third peaks at r ≈ 2.49 Å and r ≈ 2.95 Å due to backscattering of neighboring Co atoms, relate to the Co_Oct_-Co_Oct_ ≈ 2.856 Å [*N* = 4] and Co_Tet_-Co_Oct_ ≈ 3.365 Å [*N* = 8] coordination shells, respectively. The fourth peak at r ≈ 4.70 Å relates to higher-order Co-O(-Co) coordination shells (Figure 3A and Table S1). The position of the coordination peaks in the FT|*k*^3^χ(*k*)| spectra and of their corresponding Wavelet-Transform (WT) spectra shown in Figure 3B does not change among the different Co_3_O_4_ oxides. However, both the FT|*k*^3^χ(*k*)| and WT spectra exhibit a decrease in the magnitude of the Co-O, Co_Oct_-Co_Oct_, Co_Tet_-Co_Oct_ and higher Co-Co(-O) coordination peaks. The relative amplitude decay in the FT|*k*^3^χ(*k*)| and WT spectra is correlated with a decrease in the coordination number (*N*), or an increase in the mean-square disorder parameter σ^2^, which arises from static structural disorder from crystal defects due to slightly different interatomic distances in the same coordination shell. Therefore, the weakened relative amplitudes of high coordination peaks in the FT|*k*^3^χ(*k*)| and WT spectra offer an indication of the extent of crystalline short-to-medium-range order around the cobalt centers.

The Co_3_O_4_ oxides obtained from the standard synthesis, and those synthetized at 30 min holding and at 10 min ramping time, respectively, display FT|*k*^3^χ(*k*)| spectra similar to those of Co_3_O_4_ synthesized at lower Co(OAc)_2_ concentrations of 0.9 and 1.2 mmol (Figure S4). As shown in Figures 3A,B, Figure S4 and Table S1, the interatomic distances and the atomic coordination numbers *N* of those oxides remain the same, showing that their atomic short-range order is very similar regardless of the variations in the synthetic parameters, and that they exhibit higher crystallinity among the series of as-synthesized Co_3_O_4_ oxides. While the main interatomic distances and *N*-values for Co_3_O_4_ oxides synthesized at lower temperatures of 160 and 140 °C remain quite the same, those oxides show an increase in the mean-square static disorder σ^2^ (Table S1). This suggests that these oxides have slightly increased local disorder, pointing to the synthesis temperature as a crucial parameter for controlling the local order and the degree of crystallization of the bulk spinel structure. The static local-disorder σ^2^ increases more remarkably for Co_3_O_4_ oxides synthesized at H_2_O_2_ concentrations of 1.5 and 3 mL (Figures 3A,B, Figure S4 and Table S1), which indicates increased local disorder and lower degrees of crystallinity in those oxides. The latter may arise from their formation process: when adding H_2_O_2_ to the initial suspension (Co(OAc)_2_·4H_2_O + 1.5 mL H_2_O + 25% NH_3_), some divalent Co^2+^ cations are replaced by trivalent Co^3+^ species. The anions in suspension are then intercalated into the interlayer space to compensate the extra positional charge inferred by Co^3+^ cations, thus leading to the formation of a hydrotalcite-type cobalt compound (Yang et al., 2007). Thereafter, upon hydrothermal treatment at 180 °C, this intermediate hydrotalcite-cobalt compound is converted into spinel-type Co_3_O_4_ (Amiri et al., 2011). This structural transformation could induce increased local disorder in the oxides synthesized in the presence of H_2_O_2_ as oxidant. This observation agrees with the results from PXRD and Raman spectra, where Co_3_O_4_ oxides synthesized at H_2_O_2_ concentrations of 1.5 and 3 mL show broadened diffraction and Raman peaks due to the local structural dispersion of the Co and O atoms in the spinel structure (Figure 1).

**Figure 4:**
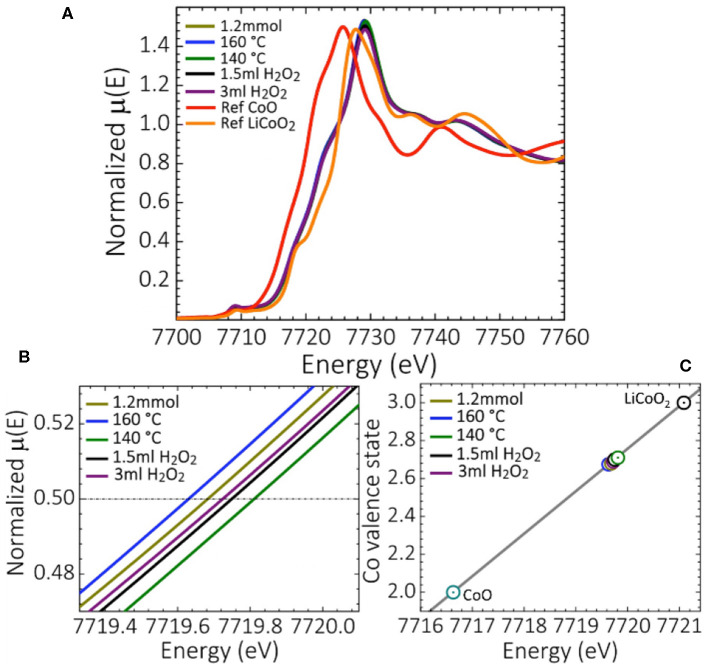
**(A)** XANES spectra of selected synthesized Co_3_O_4_ oxides, and reference compounds Co^II^O and LiCo^III^O_2_, **(B)** zoom of the Co absorption *K*-edge of the normalized XANES spectra, and **(C)** the average Co valence state determined from the XANES spectra.

X-ray absorption near edge structure (XANES) spectra were recorded to obtain further insight into the electronic properties and main oxidation states of Co in the synthesized oxides. Figure 4A shows the XANES spectra for oxides synthesized at H_2_O_2_ quantities of 3 and 1.5 mL, low temperatures of 140 and 160 °C and Co(OAc)_2_ concentrations of 1.2 mmol vs. those of the reference compounds Co^II^O and LiCo^III^O_2_. Co_3_O_4_ oxides obtained from standard synthesis, and those synthesized at 30 min holding and at 10 min ramping time displayed XANES spectra similar to those of Co_3_O_4_ synthesized at lower Co(OAc)_2_ concentrations of 0.9 and 1.2 mmol (Figure S5). The Co *K*-edge absorption edge energy of the different Co_3_O_4_ oxides is located at ≈ 7719.64–7719.82 eV (Figures 4A,B), suggesting that the oxidation state of Co is quite the same for all the synthesized Co_3_O_4_ oxides. From the linear dependence of the Co *K*-edge position at the energy corresponding to μ(*E*) ≈ 0.5 of the normalized XANES spectra of Co_3_O_4_ and reference oxides Co^II^O, LiCo^III^O_2_, the main oxidation state of Co was calculated as 2.65 (Figure 4C). This agrees with the main cobalt oxidation state of a normal Co_3_O_4_ spinel structure with 8 Co^2+^ cations located in tetrahedral and 16 Co^3+^ cations located in octahedral sites, respectively. The slight changes in the white line intensity at ≈ 7729.57 eV (Figure 4A), further indicates the existence of local disorder and a slightly different density of unoccupied Co *d*-states for Co_3_O_4_ oxides synthesized with H_2_O_2_ concentrations of 1.5 and 3 mL. This, as previously discussed, could be due to the structural transformations induced when using H_2_O_2_ as oxidant, and to some extent be caused by a charge imbalance due to the interaction of Co^2+^-Co^3+^ species (Dau et al., 2003).

**Figure 5:**
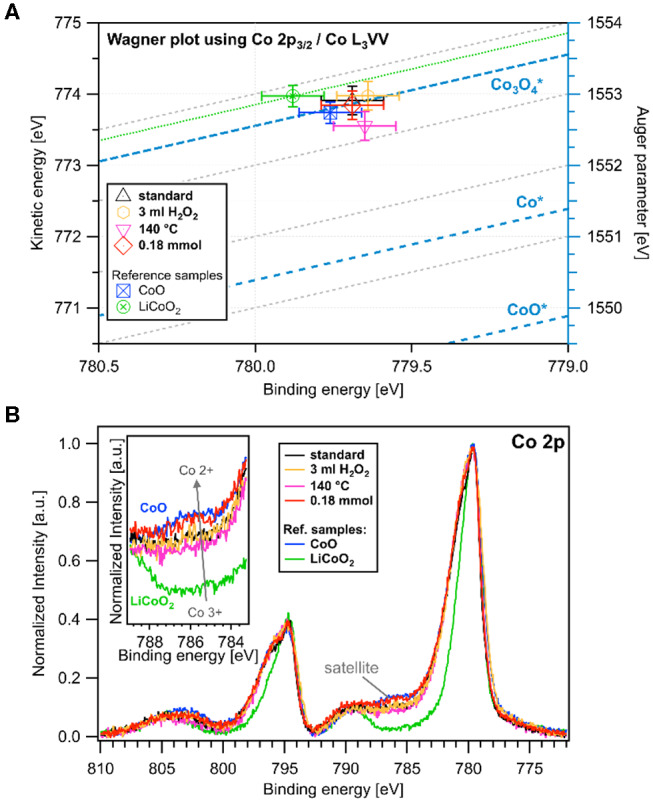
**(A)** Wagner plot of the four most different samples with the kinetic energy of the Co L_3_VV Auger electron and Co 2p_3/2_ core level binding energy with Co*, CoO*, and Co_3_${\text{O}}_{4}^{*}$ references obtained from NIST database.^50^ **(B)** XPS spectra of the Co 2p peaks of the same samples and of Co^II^O and LiCo^III^O_2_ reference compounds (inset: satellite of the Co 2p_3/2_ peak).

X-ray photoelectron spectroscopy was conducted on the standard, 3 mL H_2_O_2_, 140 °C and 0.18 mmol samples, the latter corresponding to the three synthesis conditions deviating most widely from the standard protocol. The Co 2p spectra are shown in Figure 5B with similar binding energies for all samples of ≈ 780 eV for the Co 2p_3/2_ and ≈ 795 eV for the Co 2p_1/2_ peak, which corresponds to literature values (Linstrom, 1997). The spectra of the reference compounds Co^II^O and LiCo^III^O_2_ are shown in the same graph, indicating an increase in surface oxidation state in the order 0.18 mmol < standard ≈ 3 mL H_2_O_2_ < 140 °C, which is in accordance with the XANES data shown in Figures 4A–C. The oxidation states were determined from the intensity of the shake-up satellite at 785–786 eV (inset in Figure 5B), which originates exclusively from Co^2+^, since the binding energies of Co^2+^ and Co^3+^ are too close to be distinguished (Yang et al., 2010). For this reason, a Wagner plot containing the kinetic energies of the Co L_3_VV Auger electrons vs. the corresponding binding energies of the Co 2p_3/2_ core level photoelectrons is shown in Figure 5A. Wagner plots facilitate chemical state analysis, by illustrating shifts in photoelectron lines and X-ray excited Auger electron lines as well as the modified Auger parameter (AP) which is defined as the sum of the Auger electron kinetic energy and the corresponding core-level photoelectron binding energy (Wagner, 1972). Due to its insensitivity to static charging and variations in the energy scale calibration the AP is particularly useful to compare spectra of insulating samples with literature data and spectra acquired with different instruments. Reference values for the modified AP for CoO, Co and Co_3_O_4_ are indicated in Figure 5A as diagonal blue lines (Linstrom, 1997). The APs of the as-synthesized Co_3_O_4_ samples are in agreement with previous values reported in literature, while only the parameter of the sample synthesized at low temperature (140 °C) is somewhat lower but still within the error range.

**Figure 6:**
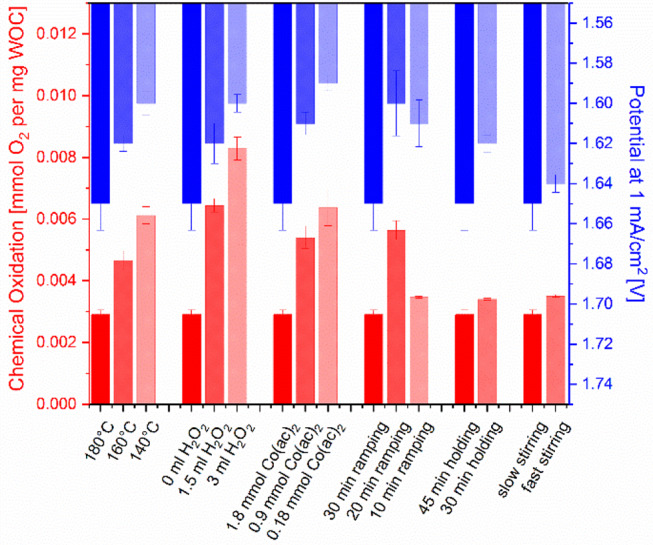
Water oxidation activity (chemical: red, electrocatalytic: blue) of the spinel samples synthesized by varying a single parameter of the standard synthesis method (dark red bar at the left of each triad). Chemical oxidation was assessed with 146 mM cerium(IV) ammonium nitrate (CAN) and the electrocatalytic activity was compared by the potentials vs. RHE at 1 mA/cm^2^ in 1 M KOH.

The obtained materials were further compared with respect to their water oxidation activity. Water oxidation tests were performed using two different methods, namely chemical and electrocatalytic water oxidation. In the first method, the water oxidation activity was assessed using cerium ammonium nitrate (CAN) which is a standard oxidant for water oxidation with a redox potential of 1.75 V vs. NHE (Patterson, 1939). The amount of formed oxygen was quantified with a luminescent dissolved oxygen electrode (Figure S6) and is shown as a function of the catalyst amount in Figure 6. The dark red bar at the beginning of each group represents the reference yield obtained from Co_3_O_4_ synthesized with standard parameters. Obviously, any change of synthesis parameters in the standard protocol leads to a better oxygen evolution. For the first three parameters, i.e., temperature, H_2_O_2_ amount and Co-precursor concentration, the oxygen concentration shows an upward trend whereas for the ramping time an optimum was obtained for 20 min. With shorter reaction (holding) time and faster stirring speed the activity was only marginally increased.

Electrocatalytic measurements were performed with a standard three electrode setup using Ag/AgCl as reference and Pt as counter electrode in alkaline environment. The use of Pt counter electrodes was recently under debate since it was shown that metal traces may dissolve in the electrolyte and influence the activity of water reduction catalysts (Cherevko et al., 2014; Chen et al., 2017; Wei et al., 2019). However, this is not applicable for the present water oxidation system, because Pt does not produce any Faradaic current in this region (see Figure S11) and the measurements were performed at pH 14 where Pt dissolution is minimized (Wei et al., 2019). Furthermore, the goal of this study is an internal comparison between spinel-type Co_3_O_4_ catalysts emerging from tuned synthetic protocols with standard electrochemical measurement techniques, rather than the discussion of absolute performance values. To this end, stepwise chronoamperometry from 0.45 V to 0.65 V vs. Ag/AgCl was performed. To obtain the bare Faradaic current without diffusion current, the current density value after 5 min holding at the same potential was considered and plotted against the applied potential (Figure S7). Electrochemical impedance spectroscopy (EIS) measurements, showing lower resistivity for Co_3_O_4_ synthesized at 140 °C compared to Co_3_O_4_ synthesized at 180 °C (standard synthesis) and long-term chronoamperometry measurements are provided in Figures S12, S13 and Table S2.

To facilitate comparison between the samples, the potential at 1 mA/cm^2^ current density is shown at the left of each triad in Figure 6 (dark blue bars). Compared to our abovementioned study where the influence of substantially different synthetic methods on the electrochemical activity was rather marginal, the present variations in electrocatalytic performance after changing a single synthetic parameter are somewhat higher and display a trend. In contrast, parameter-dependent chemical water oxidation activity differences were slightly smaller compared to the previous study covering a wider range of methods (Figure S8) (Reith et al., 2019). Notably, closely related trends for water oxidation activity were obtained by the two different test methods. Both chemical and electrochemical activity could be increased by either shorter synthesis times, addition of hydrogen peroxide or lower precursor concentrations. The optimal ramping time was found to be 20 min. Shorter synthesis holding times had a more productive influence on the electrocatalytic performance than on the chemical oxidation activity, and stirring speed was the least important parameter in both assays.

**Figure 7:**
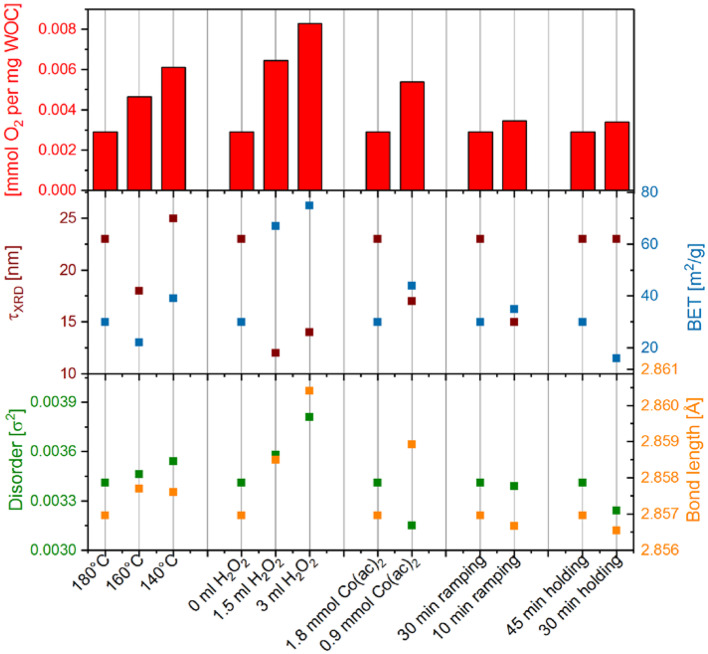
Chemical water oxidation activities of the different Co_3_O_4_ samples **(top)** compared with their respective crystallite size τ_XRD_ and BET surface area **(middle)**, as well as, to their mean-square disorder parameter σ^2^ and Co_Oct_-Co_Oct_ bond length determined from EXAFS fitting **(bottom)**.

The observed water oxidation activities do not correlate exclusively with the surface area or crystallite size of the Co_3_O_4_ samples, as seen from comparison with calculated crystallite sizes and BET-determined surface areas (Figure 7). The errors in BET and τ_XRD_ are in the range of 1-5 % (0.65 m^2^g^−1^ for BET, determined by fourfold measurement of commercial Co_3_O_4_ with similar crystallite size to most of the here investigated samples and 0.2–1 nm for τ_XRD_, determined from error propagation from PXRD peak FWHM determination). Furthermore, no correlation to any determined material parameter could be found for surface normalized activities (Figure S9). In light of the key role of the HO-Co_2_(μ-O/OH)_2_-OH edge site on Co_3_O_4_ assisted water oxidation revealed by Zhang et al. (2014a), we tentatively plotted non normalized chemical water oxidation activities vs. the bond length and disorder of Co_Oct_-Co_Oct_ as determined by EXAFS fitting (cf. Figure 7 is below). Error estimations for the disorder parameter σ^2^ and bond lengths are given in Table S1 as significant decimal digits. Other than for the other structural parameters (Co-O and Co_Oct_-Co_Tet_, see Table S1 and Figure S10), a trend was about to emerge here, but more precise data are required to establish a truly significant correlation. In comparison, other materials characteristics such as oxidation state and crystallinity did not display any clear correlations with the water oxidation activity (Figure S10). As an overall trend, however, smaller particles with larger surface area as well as lower sample crystallinity with higher disorder result in higher water oxidation activities.

## Conclusions

The present study demonstrates new insights into the importance of synthetic parameters in microwave-hydrothermal synthesis. This synthetic approach is a tunable and rapid method for the production of nanoscale spinel-type Co_3_O_4_ water oxidation catalysts, therefore holding great potential for industrial applications. Starting from a standard microwave protocol, the influence of six key synthetic parameters on the properties of the resulting cobalt oxide samples was first screened. In particular, shorter ramping times and addition of hydrogen peroxide were shown to reduce the crystallite and particle size. The crystallinity of the Co_3_O_4_ catalysts was decreased most significantly by hydrogen peroxide addition and by lower synthesis temperatures. Therefore, hydrogen peroxide addition was found to be the most influential synthesis parameter, even though the bulk cobalt oxidation state of the emerging spinels was not notably influenced as shown by XANES investigations.

The obtained Co_3_O_4_ spinels were characterized with respect to their synthesis parameters using a wide range of analytical methods, including PXRD, BET, XAS, XPS, and Raman spectroscopy as well as electron microscopy. Next, they were compared with respect to their chemical and electrocatalytic water oxidation activity. Significant changes in the respective water oxidation activities were observed as a result of the synthetic parameter variations. In particular, H_2_O_2_ addition during synthesis yielded much more active catalysts. Furthermore, lower synthesis temperature, cobalt precursor concentrations and shorter ramping times exerted a beneficial influence on the oxygen evolution performance. Stirring speed and holding time were found to be the least impactful parameters.

Correlations of the observed activity changes with the detailed materials parameters of the Co_3_O_4_ spinels indicate that not only the surface area and the crystallite size, but possibly also structural parameters, such as disorder and Co_Oct_-Co_Oct_ bond distances as present in the surface edge-site motif determine the water oxidation activity. These results illustrate the key importance of synthetic parameter tuning in heterogeneous catalyst production. Therefore, systematic explorations of the synthetic parameter space along with insight into the underlying mechanisms of catalyst formation and activity are indispensable for informed and efficient design. Future strategies may thus combine rapid machine learning parameter screening with well-designed *in situ* monitoring experiments.

## Experimental

### Synthetic Procedure

All Co_3_O_4_ samples were obtained from microwave-hydrothermal synthesis. Co(CH_3_COO)_2_·4H_2_O (Sigma-Aldrich, reagent grade) was dissolved in 15 mL water and the pH was adjusted to 11 by addition of aqueous ammonia (25% NH_3_, Merck, GR for analysis). The dispersion was poured into a 95 mL CEM Omni Teflon vessel equipped with a stirring bar and sealed accordingly. Always, two reactions were conducted simultaneously and temperature and pressure were monitored for the reaction in the reference vessel. The reaction was temperature controlled, i.e., the microwave power was adjusted according to the reference vessel temperature. The reaction mixture was heated to 180 °C in 30 min (referred to as ramping time) and kept at this temperature for 45 min (referred to as holding time), followed by a cooling period of ~30 min, followed by separation of the product by centrifugation. The samples were air-dried at 80 °C prior to further characterization. The parameters were varied as summarized in Table 2 and the samples were labeled accordingly. The standard precursor concentration of 1.8 mmol was adapted from our previous studies in order to guarantee optimal salt loads and filling degrees for the microwave-hydrothermal system to ensure homogeneous and reproducible reaction conditions which warrant comparability to preceding results.

### Catalytic Measurements

Electrocatalytic measurements were carried out in a standard three-electrode setup with an Ag/AgCl (Sigma-Aldrich glass reference electrode Ag/AgCl with 3 M KCl) as reference- and a Pt-foil as counter electrode. The working electrode was prepared by first dispersing the WOC in water (2 mg in 100 mL) and 40 mL of this dispersion were drop casted on 1 cm^2^ FTO (Aldrich, ~7 Ω/sq). After 30 min drying at 80 °C, Nafion solution (10 mL, 1wt-% diluted from 5 wt-%, Nafion™ perfluorinated resin solution, Sigma-Aldrich) was drop-casted on the surface to fix the WOC. For the measurements, 1 M KOH was used as electrolyte. A Bio-Logic SAS SP-150 potentiostat was used. The standard measurement protocol consisted of three steps: (1) Cyclic voltammetry (CV) was measured from 0 to 0.7 V vs. Ag/AgCl with 0.005 mV step size. For data evaluation, only the second cycles were compared to each other. (2) Stepwise chronoamperometry increasing the potential from 0.45 V to 0.65 V in 0.01 V steps and holding for 5 min. The stabilized current at the end of each step was used for further evaluation to eliminate diffusion currents. (3) Chronoamperometry in which a potential of 0.6 V vs. Ag/AgCl was applied for 2 h for stability testing. For EIS and long-term chronoamperometry studies in 1.0 M KOH, 2 mg of the obtained cobalt oxide powders were dispersed in 100 μL of 2% Nafion (ethanol solution) and sonicated for 15 min. A total of 40 μL of the suspension was drop-cast on the carbon cloth (Toray Carbon Paper, TGP-H-60, 1 cm^2^) and kept at 60 °C for 15 min. Measurement details are provided in the Supplementary Material.

To evaluate the chemical water oxidation activity of the catalysts, the following standard CAN method was applied: Prior to addition of WOC, CAN (2 g) was dissolved in milli-Q water (40 mL) and the solution was degassed with argon. After subsequent addition of WOC (2 mg) the oxygen evolution was recorded for 45 min while stirring the solution, using a luminescent dissolved oxygen sensor (LDO). For data evaluation, only the peak concentration was used. A Hach HQ40d multimeter with LDO 101 sensor was used for these measurements.

### Materials and Methods

The Co_3_O_4_ spinels were characterized by powder X-ray diffraction (PXRD) using a STOE STADI P diffractometer in transmission mode (Ge monochromator, Mo K_α1_ radiation). The surface area was measured using Brunauer–Emmett–Teller (BET) on a Quadrasorb SI machine in N_2_-adsorption mode. Samples were degassed at 100 °C overnight under vacuum prior to the measurements. Raman spectra were measured on a Renishaw inVia Qontor confocal Raman microscope with a diode laser (785 nm). SEM images were taken with a Zeiss SUPRA 50VP SEM equipped with a Schottky field emitter and an in lens secondary electron (SE) detector. An acceleration voltage of 10 kV and a working distance of 5.4–6 mm were applied. TEM images were acquired using a JEOL JEM-1400 Plus microscope. The camera was a JEOL CCD Ruby (8 M pixel) and the electron beam source a LaB_6_ crystal operated at 120 kV. HR-TEM images were obtained from a FEI Tecnai F30 FEG device, equipped with a CCD Gatan 794 MultiScan Camera and a Schottky emitter (300 kV). X-ray photoelectron spectroscopy (XPS) was conducted on a Physical Electronics (PHI) Quantum 2000 spectrometer. The monochromatic Al-K_α_ radiation was generated from an electron beam (15 kV, 35.8 W). For energy scale calibration, Au and Cu reference samples were used. The measurements were carried out at 10^−8^ mbar, with an electron take off angle of 45° and a pass-energy of 23.5 eV. A low energy electron source was used for charge compensation throughout the measurements. The alignment of the acquired spectra was performed using the main (CC) component of the C1s core level emission. The modified Auger parameter was calculated by adding the kinetic energy of the CoL3VV and Co 2p_3/2_ binding energies.

X-ray absorption spectroscopy (XAS) spectra were recorded at the Co *K*-edge on solid powder samples dispersed in cellulose. Measurements of the synthesized Co_3_O_4_ samples and reference oxides Co^II^O and LiCo^III^O_2_ were carried out at the SuperXAS beamline at the Swiss Light Source (SLS), Paul Scherrer Institute (PSI) Villigen, Switzerland. Spectra were recorded using quick-scanning (QEXAFS) acquisition data protocols using a three-ionization chamber configuration in transmission mode and a 5-element Silicon Drift Detector. For energy calibration, the spectrum of a metal Co foil was measured simultaneously at the second ionization chamber. The X-ray beam was collimated using a Si coated mirror and the energy was scanned using a channel-cut Si[111] monochromator. A Rh coating toroidal mirror was used after the monochromator to focus the incident X-rays with a spot size of 400 × 200 μm^2^ on the samples and a photon flux of 5.0 × 10^11^ photons/s. The measured EXAFS spectra *k*^3^χ(*k*) were extracted by standard data reduction, absorption edge energy calibration and background subtraction as implemented in ATHENA (Ravel and Newville, 2005). The spectra were reduced into the range Δ*k* ≈ 3–14 Å^−1^ and Fourier transform to FT|*k*^3^χ(*k*)| into the real-space interval of ΔR ≈ 0–6 Å. To calculate main values for the interatomic distances, atomic coordination numbers (*N*), and Debye-Waller factors (σ^2^) non-linear least-squares fitting of the FT|*k*^3^χ(*k*)| spectra was carried out with ARTEMIS using atomic clusters of Co_3_O_4_ (ICSD code 27498), generated with ATOMS as implemented in IFEFFIT (Ravel and Newville, 2005). The amplitudes and phases shift for single and multiple scattering paths were calculated using FEFF6 (Ankudinov et al., 1998).

Samples with low signal to noise ratio due to low theoretical yields from downscaled precursor concentrations were not included into the SI for the sake of clarity.

## Data Availability Statement

The raw data supporting the conclusions of this article will be made available by the authors, without undue reservation.

## Author Contributions

KL and GP conceived the research. KL prepared the samples, carried out PXRD, SEM, HR-TEM, chemical, and electrocatalytic measurements and data analyses. KL and LR carried out Raman and TEM measurements. KL and CT performed XAS experiments and data analyses. SS performed XPS experiments and analyses. KL, CT, SS, and GP wrote the manuscript. All authors have given approval to the final version of the manuscript.

## Conflict of Interest

The authors declare that the research was conducted in the absence of any commercial or financial relationships that could be construed as a potential conflict of interest.

## References

[B1] Amiri S. E. H. Vaezi M. R. Kandjani A. E. (2011). A comparison between hydrothermally prepared Co_3_O_4_ via H_2_O_2_ assisted and calcination methods. J. Ceram. Process. Res. 327–331.

[B2] Ankudinov A. L. Ravel B. Rehr J. J. Conradson S. D. (1998). Real-space multiple-scattering calculation and interpretation of x-ray-absorption near-edge structure. Phys. Rev. B 58, 7565–7576. 10.1103/PhysRevB.58.7565

[B3] Artero V. Chavarot-Kerlidou M. Fontecave M. (2011). Splitting water with cobalt. Angew. Chem. 50, 7238–7266. 10.1002/anie.201007987 21748828

[B4] Bergmann A. Martinez-Moreno E. Teschner D. Chernev P. Gliech M. Araújo J. F.. (2015). Reversible amorphization and the catalytically active state of crystalline Co_3_O_4_ during oxygen evolution. Nat. Commun. 6:8625. 10.1038/ncomms9625 26456525 PMC4633955

[B5] Bilecka I. Elser P. Niederberger M. (2009). Kinetic and thermodynamic aspects in the microwave-assisted synthesis of ZnO nanoparticles in benzyl alcohol. ACS Nano 3, 467–477. 10.1021/nn800842b 19236087

[B6] Bilecka I. Niederberger M. (2010). Microwave chemistry for inorganic nanomaterials synthesis. Nanoscale 2:1358. 10.1039/b9nr00377k 20845524

[B7] Chen R. Yang C. Cai W. Wang H.-Y. Miao J. Zhang L. (2017). Use of platinum as the counter electrode to study the activity of nonprecious metal catalysts for the hydrogen evolution reaction. ACS Energy Lett. 2, 1070–1075. 10.1021/acsenergylett.7b00219

[B8] Cherevko S. Zeradjanin A. R. Keeley G. P. Mayrhofer K. J. J. (2014). A comparative study on gold and platinum dissolution in acidic and alkaline media. J. Electrochem. Soc. 161, H822–H830. 10.1149/2.0881412jes

[B9] Chu S. Li W. Yan Y. Hamann T. Shih I. Wang D. (2017). Roadmap on solar water splitting: current status and future prospects. Nano Futures 1:22001 10.1088/2399-1984/aa88a1

[B10] Chua C. S. Ansovini D. Lee C. J. J. Teng Y. T. Ong L. T. Chi D.. (2016). The effect of crystallinity on photocatalytic performance of Co_3_O_4_ water-splitting cocatalysts. Phys. Chem. Chem. Phys. 18, 5172–5178. 10.1039/C5CP07589K 26805577

[B11] Conrad F. Bauer M. Weyeneth S. Zhou Y. Hametner K. Günther D. (2013). Hierarchically structured copper gallium spinels through microwave hydrothermal methods. Solid State Sci. 24, 125–132. 10.1016/j.solidstatesciences.2013.06.016

[B12] Conrad F. Massue C. Kühl S. Kunkes E. Girgsdies F. Kasatkin I.. (2012). Microwave-hydrothermal synthesis and characterization of nanostructured copper substituted ZnM_2_O_4_ (M = Al, Ga) spinels as precursors for thermally stable Cu catalysts. Nanoscale 4, 2018–2028. 10.1039/c2nr11804a 22327266

[B13] Conrad F. Zhou Y. Yulikov M. Hametner K. Weyeneth S. Jeschke G. (2010). Microwave-hydrothermal synthesis of nanostructured zinc-copper gallates. Eur. J. Inorg. Chem. 2010, 2036–2043. 10.1002/ejic.200901169

[B14] Cordeiro P. V. O. Carvalho N. M. F. (2018). Water oxidation reaction catalyzed by Co_3_O_4_ treated with organic compounds. Ind. Eng. Chem. Res. 57, 11259–11264. 10.1021/acs.iecr.8b01962

[B15] Dau H. Liebisch P. Haumann M. (2003). X-ray absorption spectroscopy to analyze nuclear geometry and electronic structure of biological metal centers-potential and questions examined with special focus on the tetra-nuclear manganese complex of oxygenic photosynthesis. Anal. Bioanal. Chem. 376, 562–583. 10.1007/s00216-003-1982-2 12802563

[B16] Dong H. Kuzmanoski A. Wehner T. Müller-Buschbaum K. Feldmann C. (2016). Microwave-assisted polyol synthesis of water dispersible red-emitting Eu^3+^-modified carbon dots. Materials 10, 1–10. 10.3390/ma10010025 28772378 PMC5344616

[B17] Gao R. Yang Z. Zheng L. Gu L. Liu L. Lee Y.. (2018). Enhancing the catalytic activity of Co_3_O_4_ for Li–O_2_ batteries through the synergy of surface/interface/doping engineering. ACS Catal. 8, 1955–1963. 10.1021/acscatal.7b0356630788960

[B18] Gawali S. R. Gandhi A. C. Gaikwad S. S. Pant J. Chan T.-S. Cheng C.-L.. (2018). Role of cobalt cations in short range antiferromagnetic Co_3_O_4_ nanoparticles: a thermal treatment approach to affecting phonon and magnetic properties. Sci. Rep. 8:249. 10.1038/s41598-017-18563-9 29321560 PMC5762665

[B19] Grzelczak M. Zhang J. Pfrommer J. Hartmann J. Driess M. Antonietti M. (2013). Electro- and photochemical water oxidation on ligand-free Co_3_O_4_ nanoparticles with tunable sizes. ACS Catal. 3, 383–388. 10.1021/cs3007523

[B20] Hadjiev V. G. Iliev M. N. Vergilov I. V. (1988). The Raman spectra of Co_3_O_4_. J. Phys. C: Solid State Phys. 199–201. 10.1088/0022-3719/21/7/007

[B21] Hilaire S. Süess M. J. Kränzlin N. Bienkowski K. Solarska R. Augustynski J. (2014). Microwave-assisted nonaqueous synthesis of WO_3_ nanoparticles for crystallographically oriented photoanodes for water splitting. J. Mater. Chem. A 2, 20530–20537. 10.1039/C4TA04793A

[B22] Iosub A. V. Stahl S. S. (2015). Catalytic aerobic dehydrogenation of nitrogen heterocycles using heterogeneous cobalt oxide supported on nitrogen-doped carbon. Org. Lett. 17, 4404–4407. 10.1021/acs.orglett.5b01790 26333043 PMC6040916

[B23] Koziej D. Floryan C. Sperling R. A. Ehrlicher A. J. Issadore D. Westervelt R.. (2013). Microwave dielectric heating of non-aqueous droplets in a microfluidic device for nanoparticle synthesis. Nanoscale 5, 5468–5475. 10.1039/c3nr00500c 23670701

[B24] Kuzmanoski A. Pankratov V. Feldmann C. (2015). Microwave-assisted ionic-liquid-based synthesis of highly crystalline CaMoO_4_:RE^3+^ (RE = Tb, Sm, Eu) and Y_2_Mo_4_O_15_:Eu^3+^ nanoparticles. Solid State Sci. 41, 56–62. 10.1016/j.solidstatesciences.2015.02.005

[B25] Li J. Güttinger R. Moré R. Song F. Wan W. Patzke G. R. (2017). Frontiers of water oxidation: the quest for true catalysts. Chem. Soc. Rev. 46, 6124–6147. 10.1039/C7CS00306D 28745756

[B26] Li W. Y. Xu L. N. Chen J. (2005). Co_3_O_4_ Nanomaterials in lithium-ion batteries and gas sensors. Adv. Funct. Mater. 15, 851–857. 10.1002/adfm.200400429

[B27] Linstrom P. (1997). NIST Chemistry WebBook. Washington, DC: National Institute of Standards and Technology, © 1997.

[B28] Liu Q. Chen Z. Yan Z. Wang Y. Wang E. Wang S. (2018). Crystal-plane-dependent activity of spinel Co_3_O_4_ towards water splitting and the oxygen reduction reaction. ChemElectroChem 5, 1080–1086. 10.1002/celc.201701302

[B29] Luo Y. Kong D. Luo J. Wang Y. Zhang D. Qiu K. (2014). Seed-assisted synthesis of Co_3_O_4_@α-Fe_2_O_3_ core–shell nanoneedle arrays for lithium-ion battery anode with high capacity. RSC Adv. 4:13241 10.1039/c3ra47189f

[B30] Moura A. P. Cavalcante L. S. Sczancoski J. C. Stroppa D. G. Paris E. C. Ramirez A. J. (2010). Structure and growth mechanism of CuO plates obtained by microwave-hydrothermal without surfactants. Adv. Powder Technol. 21, 197–202. 10.1016/j.apt.2009.11.007

[B31] Najafpour M. M. Pashaei B. Nayeri S. (2012). Nano-sized layered aluminium or zinc-manganese oxides as efficient water oxidizing catalysts. Dalton Trans. 41, 7134–7140. 10.1039/c2dt30353a 22565665

[B32] Najafpour M. M. Renger G. Hołynska M. Moghaddam A. N. Aro E.-M. Carpentier R.. (2016). Manganese compounds as water-oxidizing catalysts: from the natural water-oxidizing complex to nanosized manganese oxide structures. Chem. Rev. 116, 2886–2936. 10.1021/acs.chemrev.5b00340 26812090

[B33] Nüchter M. Ondruschka B. Bonrath W. Gum A. (2004). Microwave assisted synthesis – a critical technology overview. Green Chem. 6, 128–141. 10.1039/B310502D

[B34] Patterson A. L. (1939). The scherrer formula for X-ray particle size determination. Phys. Rev. 56, 978–982. 10.1103/PhysRev.56.978

[B35] Ravel B. Newville M. (2005). ATHENA, ARTEMIS, HEPHAESTUS: data analysis for X-ray absorption spectroscopy using IFEFFIT. J. Synchrotron Radiat. 12, 537–541. 10.1107/S0909049505012719 15968136

[B36] Reith L. Lienau K. Cook D. S. Moré R. Walton R. I. Patzke G. R. (2018). Monitoring the hydrothermal growth of cobalt spinel water oxidation catalysts: from preparative history to catalytic activity. Chem. Eur. J. 24, 18424–18435. 10.1002/chem.201801565 29790222

[B37] Reith L. Lienau K. Triana C. A. Siol S. Patzke G. R. (2019). Preparative history vs driving force in water oxidation catalysis: parameter space studies of cobalt spinels. ACS Omega 4, 15444–15456. 10.1021/acsomega.9b01677 31572845 PMC6761687

[B38] Rosen J. Hutchings G. S. Jiao F. (2014). Synthesis, structure, and photocatalytic properties of ordered mesoporous metal-doped Co_3_O_4_. J. Catal. 310, 2–9. 10.1016/j.jcat.2013.05.003

[B39] Scherrer P. (1918). “Bestimmung der Größe und der inneren Struktur von Kolloidteilchen mittels Röntgenstrahlen,” in Nachrichten von der Gesellschaft der Wissenschaften zu Göttingen, Mathematisch-Physikalische Klasse 98–100.

[B40] Tung C.-W. Hsu Y.-Y. Shen Y.-P. Zheng Y. Chan T.-S. Sheu H.-S.. (2015). Reversible adapting layer produces robust single-crystal electrocatalyst for oxygen evolution. Nat. Commun. 6:8106. 10.1038/ncomms9106 26315066 PMC4560826

[B41] Wagner C. D. (1972). Auger lines in x-ray photoelectron spectrometry. Anal. Chem. 44, 967–973. 10.1021/ac60314a015 22309547

[B42] Wang D. Yu Y. He H. Wang J. Zhou W. Abruña H. D. (2015). Template-free synthesis of hollow-structured Co_3_O_4_ nanoparticles as high-performance anodes for lithium-ion batteries. ACS Nano 9, 1775–1781. 10.1021/nn506624g 25602513

[B43] Wang H.-Y. Hung S.-F. Chen H.-Y. Chan T.-S. Chen H. M. Liu B. (2016b). In operando identification of geometrical-site-dependent water oxidation activity of spinel Co_3_O_4_. J. Am. Chem. Soc. 138, 36–39. 10.1021/jacs.5b10525 26710084

[B44] Wang J. Cui W. Liu Q. Xing Z. Asiri A. M. Sun X. (2016a). Recent progress in cobalt-based heterogeneous catalysts for electrochemical water splitting. Adv. Mater. 28, 215–230. 10.1002/adma.201502696 26551487

[B45] Wang R. M. Liu C. M. Zhang H. Z. Chen C. P. Guo L. Xu H. B. (2004). Porous nanotubes of Co_3_O_4_: synthesis, characterization, and magnetic properties. Appl. Phys. Lett. 85, 2080–2082. 10.1063/1.1789577

[B46] Wei C. Rao R. R. Peng J. Huang B. Stephens I. E. L. Risch M.. (2019). Recommended practices and benchmark activity for hydrogen and oxygen electrocatalysis in water splitting and fuel cells. Adv. Mater. 31:e1806296. 10.1002/adma.201806296 30656754

[B47] Wei R. Fang M. Dong G. Lan C. Shu L. Zhang H.. (2018). High-index faceted porous Co_3_O_4_ nanosheets with oxygen vacancies for highly efficient water oxidation. ACS Appl. Mater. Inter. 10, 7079–7086. 10.1021/acsami.7b18208 29406690

[B48] Yang J. Liu H. Martens W. N. Frost R. L. (2010). Synthesis and characterization of cobalt hydroxide, cobalt oxyhydroxide, and cobalt oxide nanodiscs. J. Phys. Chem. C 114, 111–119. 10.1021/jp908548f

[B49] Yang Y.-P. Liu R.-S. Huang K.-L. Wang L.-P. Liu S.-Q. Zeng W.-W. (2007). Preparation and electrochemical performance of nanosized Co_3_O_4_ via hydrothermal method. Trans. Nonferrous Met. Soc. China 17, 1334–1338. 10.1016/S1003-6326(07)60272-6

[B50] Zeng G. Caputo R. Carriazo D. Luo L. Niederberger M. (2013). Tailoring two polymorphs of LiFePO_4_ by efficient microwave-assisted synthesis: a combined experimental and theoretical study. Chem. Mater. 25, 3399–3407. 10.1021/cm400995g

[B51] Zhang G. Yang J. Wang H. Chen H. Yang J. Pan F. (2017). Co_3_O_4−δ_ quantum dots as a highly efficient oxygen evolution reaction catalyst for water splitting. ACS Appl. Mater. Inter. 9, 16159–16167. 10.1021/acsami.7b01591 28447457

[B52] Zhang M. de Respinis M. Frei H. (2014a). Time-resolved observations of water oxidation intermediates on a cobalt oxide nanoparticle catalyst. Nat. Chem. 6, 362–367. 10.1038/nchem.1874 24651205

[B53] Zhang N. Shi J. Mao S. S. Guo L. (2014b). Co_3_O_4_ quantum dots: Reverse micelle synthesis and visible-light-driven photocatalytic overall water splitting. Chem. Comm. 50, 2002–2004. 10.1039/c3cc48026g 24413340

[B54] Zhang N. Wang Y. Hao Y.-C. Ni Y.-M. Su X. Yin A.-X.. (2018). Ultrathin cobalt oxide nanostructures with morphology-dependent electrocatalytic oxygen evolution activity. Nanoscale 10, 20313–20320. 10.1039/C8NR05337E 30375608

[B55] Zhao Q. Yan Z. Chen C. Chen J. (2017). Spinels: controlled preparation, oxygen reduction/evolution reaction application, and beyond. Chem. Rev. 117, 10121–10211. 10.1021/acs.chemrev.7b00051 28745484

[B56] Zhou J. Li J. Zhang L. Song S. Wang Y. Lin X. (2018). Highly active surface structure in nanosized spinel cobalt-based oxides for electrocatalytic water splitting. J. Phys. Chem. C 122, 14447–14458. 10.1021/acs.jpcc.8b00407

[B57] Zhou M. Cai L. Bajdich M. García-Melchor M. Li H. He J. (2015). Enhancing Catalytic CO Oxidation over Co_3_O_4_ Nanowires by Substituting Co^2+^ with Cu^2+^. ACS Catal. 5, 4485–4491. 10.1021/acscatal.5b00488

