# Supplementary material for: Microwave-Hydrothermal Tuning of Spinel-Type Co3O4 Water Oxidation Catalysts
Source: Front Chem. 2020 Jun 9;8:473. doi: 10.3389/fchem.2020.00473 (PMC7296166; doi:10.3389/fchem.2020.00473)
Supplement: Supplementary file 1 [file Data_Sheet_1.pdf]

Supplementary Material

Table of Contents

I. Characterization of as-synthesized Co_3O_4 materials.

Figure S1. Raman peak centers for the different Co_3O_4 samples. S2

Figure S2. SEM images of all Co_3O_4 samples. S3

Figure S3. HR-TEM images of selected Co_3O_4 samples. S4

Figure S4. EXAFS spectra and fitting of all investigated Co_3O_4 samples. S5

Figure S5. XANES spectra of all investigated Co_3O_4 samples. S6

Table S1. EXAFS data calculated from fitting of the experimental spectra. S7

II. Catalytic measurements.

Figure S6. LDO measurement of chemical oxidation of all Co_3O_4 samples. S8

Figure S7. Chronoamperometric measurements of cobalt oxide samples. S8

Figure S8. Chemical water oxidation activity compared to previous studies. S9

Figure S9. Surface normalized chemical water oxidation activities. S9

Figure S10. EXAFS and XANES derived parameters vs. catalytic activity. S10

Figure S11. LSV of Co_3O_4 vs Pt. S11

Figure S12. Nyquist and Bode plots. S12

Table S2. Equivalent circuit and its parameters. S13

Figure S13. Long-term chronoamperometry measurements. S14

III. References. S14

I. Characterization of as-synthesized Co_3O_4 materials.

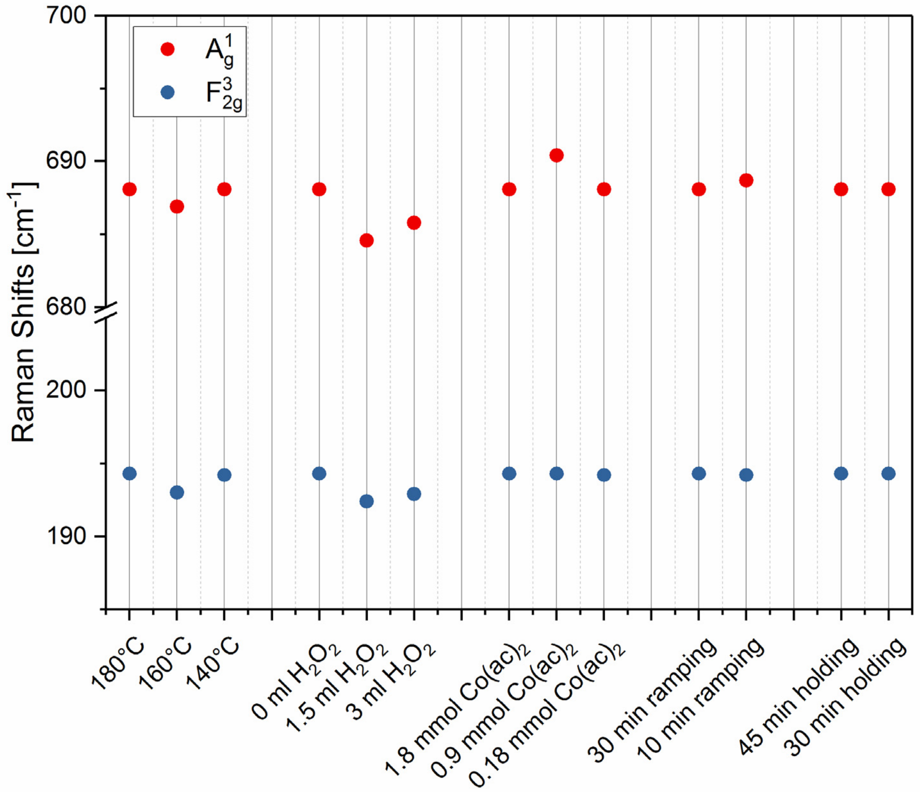

Supplementary Figure S1. Shifts in the A^1_g and F^3_{2g} Raman peak centers for the different samples.

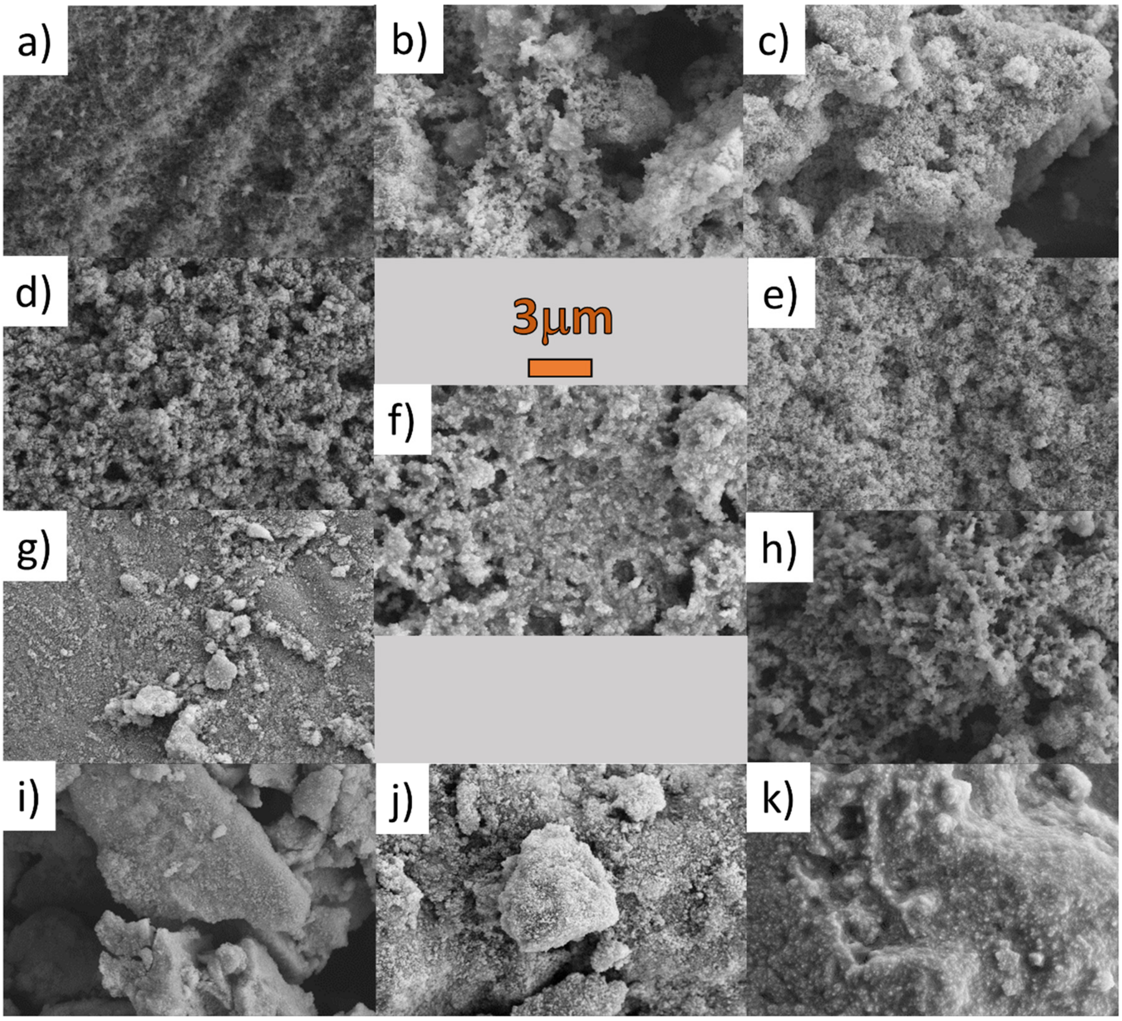

Supplementary Figure S2. SEM images of the Co_3O_4 spinel samples synthesized with single parameter variations from the standard synthesis method (f)): (a) and (b) 0.18 and 0.9 mmol cobalt precursor, respectively, (c) and (e) 140 °C and 160 °C synthesis temperature, (d) faster stirring speed, (g) and (i) addition of 1.5 and 3 mL H_2O_2 (30wt-%), (h) 30 min holding time, (j) and (k) with 20 and 10 min ramping time, respectively.

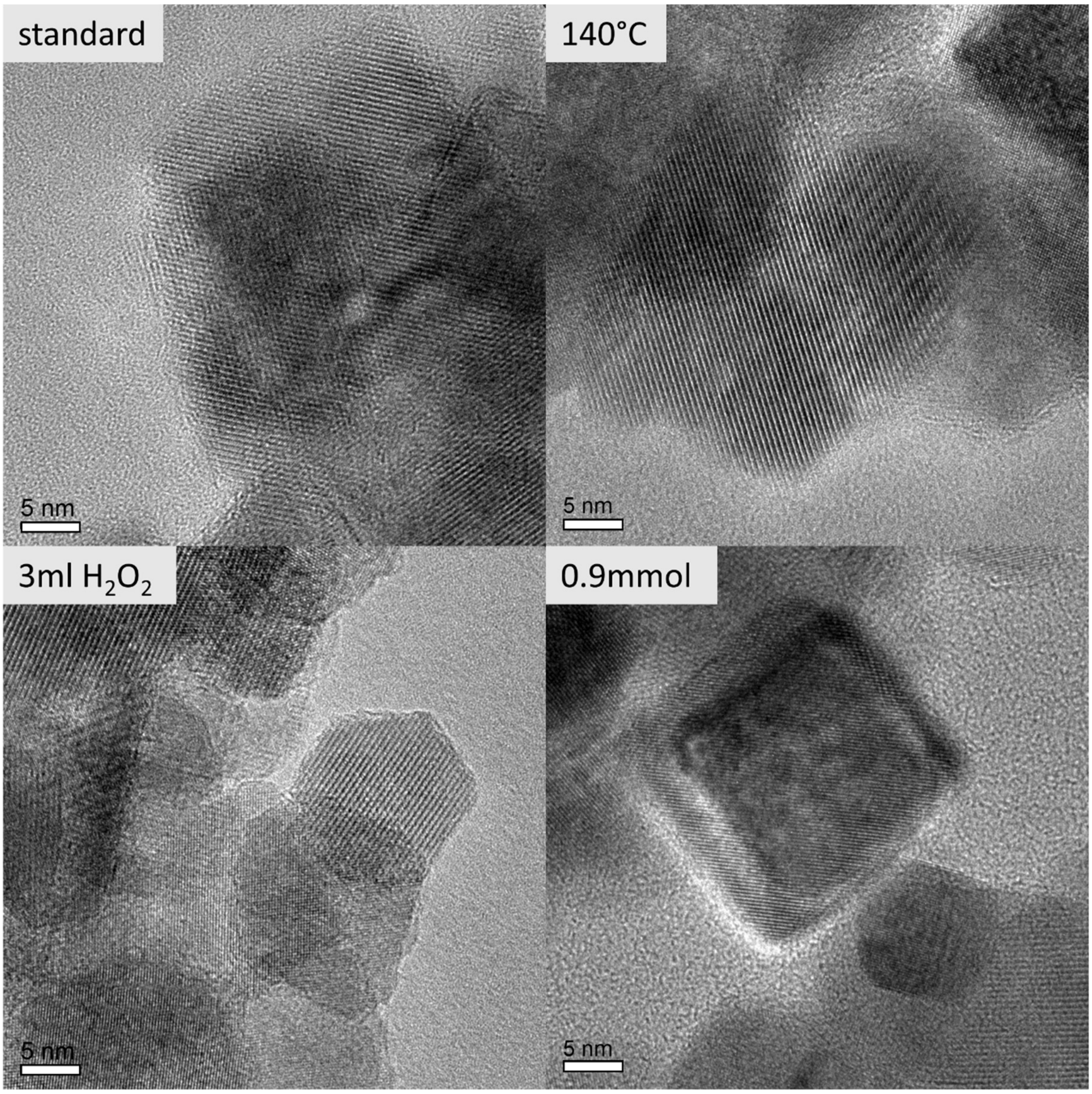

Supplementary Figure S3. High-resolution TEM images of four selected Co_3O_4 samples: (1) standard synthesis conditions, (2) sample synthesized at lower temperature (140 °C), (3) sample resulting from H_2O_2 addition (3 mL) to the synthetic mixture and (4) sample with less $Co(OAc)_2$ precursor (0.9 mmol).

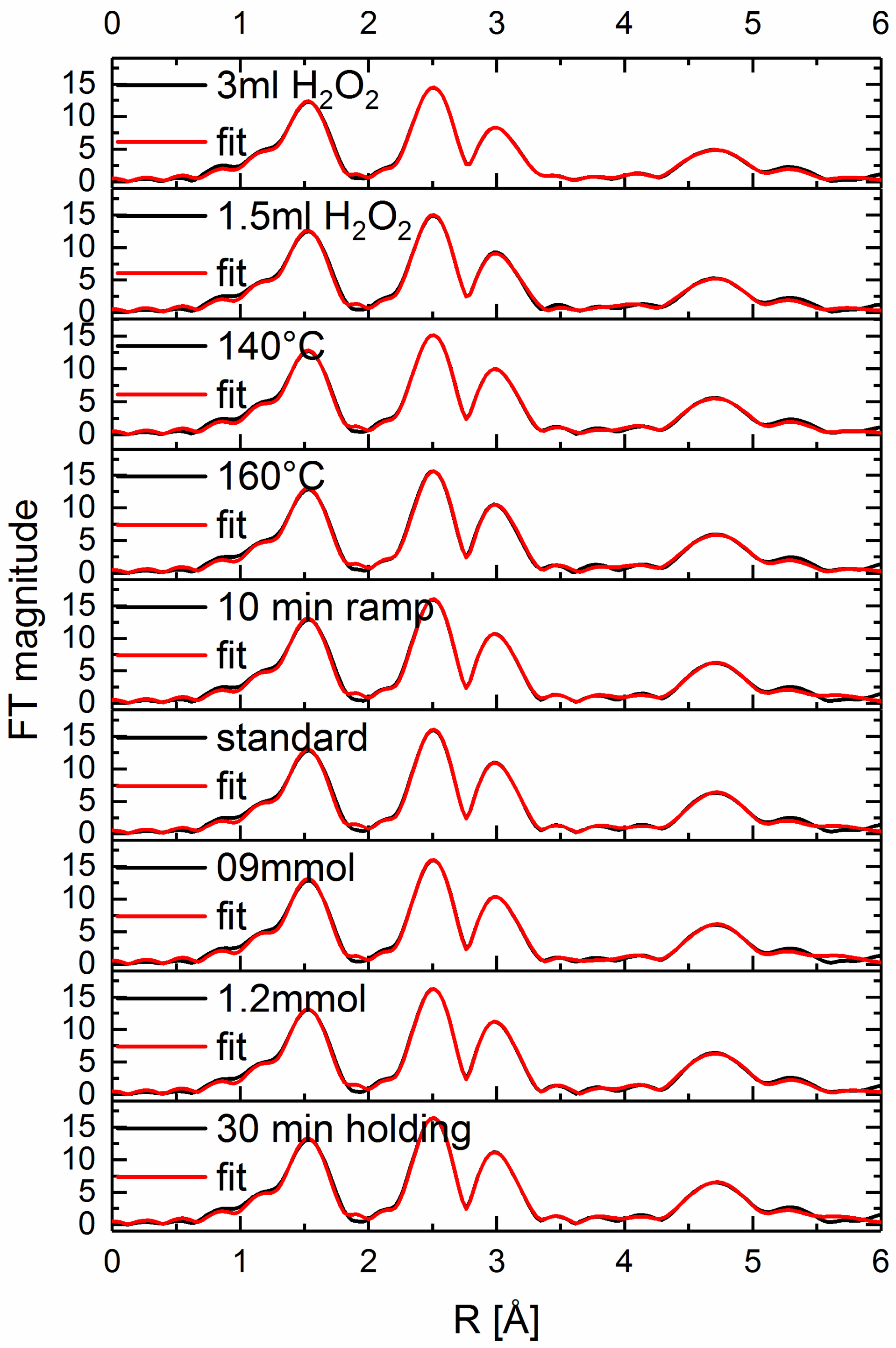

Supplementary Figure S4. Fitting of the Fourier-Transformed FT|$k^3\chi(k)$| (red) of the experimental Co *K*-edge EXAFS spectra $k^3\chi(k)$ (grey) of all measured Co_3O_4 oxides, phase uncorrected.

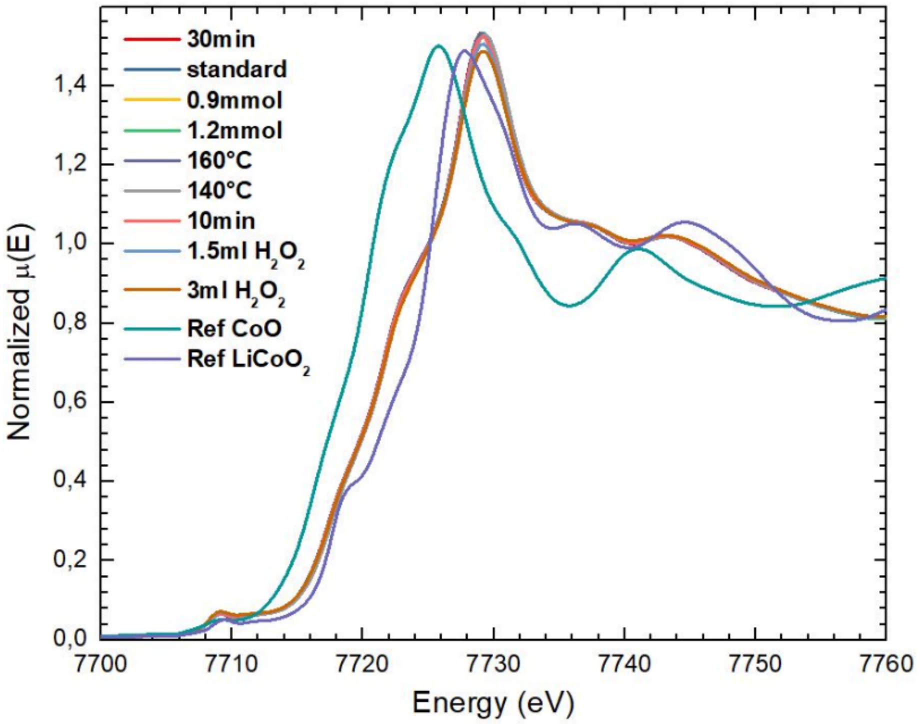

Supplementary Figure S5. XANES spectra of all synthesized Co_3O_4 oxides, vs reference compounds $Co^{II}O$ and $LiCo^{III}O_2$.

Supplementary Table S1. Main interatomic distances, atomic coordination numbers (*N*) and Debye-Waller factors (σ^2) calculated from the fitting of the experimental Co *K*-edge FT|$k^3\chi(k)$| spectra of the different synthesized spinel Co_3O_4 oxides (phase uncorrected).

Sample	Bond	*N*	σ^2	**r** (A)
**1.2 mmol Co**	Co-O	5.33	0.002(91)	1.912(7)
S_0^2≈0.752	Co_{Octa}-Co_{Octa}	4.00	0.003(25)	2.855(8)
E_0≈-1.252 eV	Co_{Tetra}-Co_{Octa}	8.00	0.006(53)	3.349(6)
	Co-Co	8.00	0.003(54)	4.962(1)
**30 min holding**	Co-O	5.33	0.002(89)	1.9124(5)
S_0^2≈0.752	Co_{Octa}-Co_{Octa}	4.00	0.003(24)	2.8565(5)
E_0≈-1.296	Co_{Tetra}-Co_{Octa}	8.00	0.008(33)	3.3671(3)
	Co-Co	8.00	0.004(37)	4.9661(3)
**Standard**	Co-O	5.33	0.003(05)	1.9127(4)
S_0^2≈0.752	Co_{Octa}-Co_{Octa}	4.00	0.003(41)	2.8569(6)
E_0≈-1.222	Co_{Tetra}-Co_{Octa}	8.00	0.008(78)	3.3658(0)
	Co-Co	8.00	0.004(63)	4.9682(2)
**10 min ramping**	Co-O	5.33	0.002(99)	1.9122(4)
S_0^2≈0.752	Co_{Octa}-Co_{Octa}	4.00	0.003(39)	2.8566(7)
E_0≈-1.203	Co_{Tetra}-Co_{Octa}	8.00	0.008(90)	3.3674(6)
	Co-Co	8.00	0.004(61)	4.9672(4)
**0.9 mmol Co**	Co-O	5.33	0.003(00)	1.9156(1)
S_0^2≈0.752	Co_{Octa}-Co_{Octa}	4.00	0.003(15)	2.8589(2)
E_0≈-0.549	Co_{Tetra}-Co_{Octa}	8.00	0.005(22)	3.3503(1)
	Co-Co	8.00	0.004(24)	4.9737(8)
**160 °C**	Co-O	5.33	0.003(00)	1.913(6)
S_0^2≈0.752	Co_{Octa}-Co_{Octa}	4.00	0.003(46)	2.857(7)
E_0≈-0.966 eV	Co_{Tetra}-Co_{Octa}	8.00	0.006(75)	3.354(8)
	Co-Co	8.00	0.004(10)	4.967(0)
**140 °C**	Co-O	5.33	0.003(10)	1.915(3)
S_0^2≈0.752	Co_{Octa}-Co_{Octa}	4.00	0.003(54)	2.857(6)
E_0≈-0.687 eV	Co_{Tetra}-Co_{Octa}	8.00	0.006(97)	3.350(9)
	Co-Co	8.00	0.004(28)	4.967(2)
**1.5 ml H_2O_2**	Co-O	5.33	0.003(23)	1.912(7)
S_0^2≈0.752	Co_{Octa}-Co_{Octa}	4.00	0.003(58)	2.858(5)
E_0≈-0.985 eV	Co_{Tetra}-Co_{Octa}	8.00	0.007(18)	3.392(1)
	Co-Co	8.00	0.005(03)	4.967(2)
**3 ml H_2O_2**	Co-O	5.33	0.003(42)	1.913(5)
S_0^2≈0.752	Co_{Octa}-Co_{Octa}	4.00	0.003(81)	2.860(4)
E_0≈-0.959 eV	Co_{Tetra}-Co_{Octa}	8.00	0.007(97)	3.361(8)
	Co-Co	8.00	0.006(18)	4.963(7)

II. Catalytic measurements.

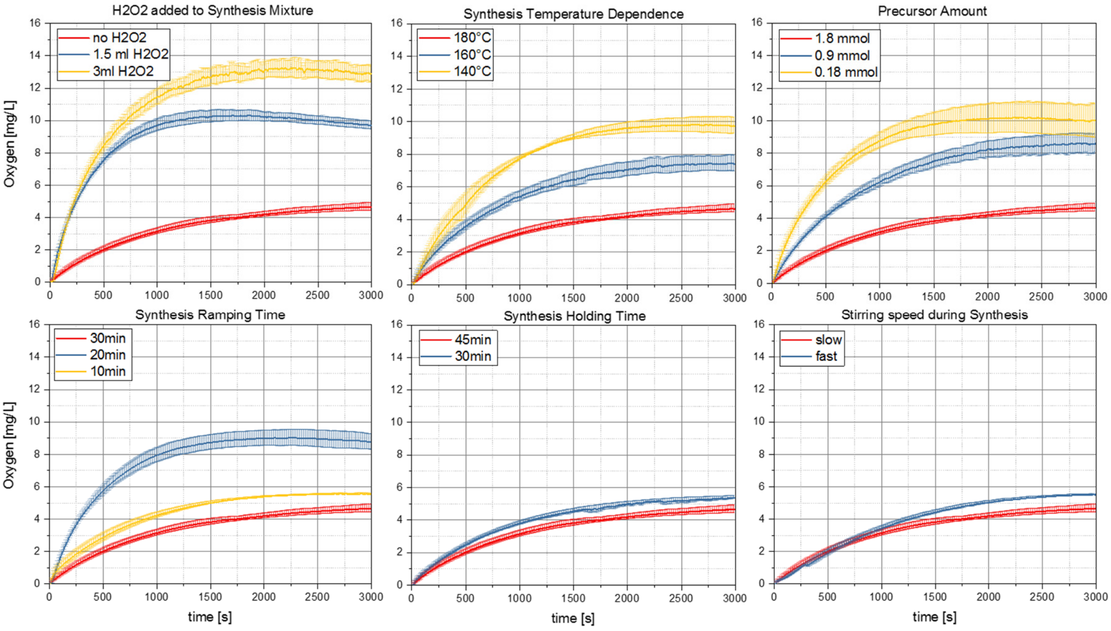

Supplementary Figure S6. Chemical oxidation with cerium ammonium nitrate (CAN, 1 g in 20 mL water) and the differently synthesized Co_3O_4 catalysts (1 mg). Oxygen concentration was continuously measured with a luminescent dissolved oxygen electrode (LDO).

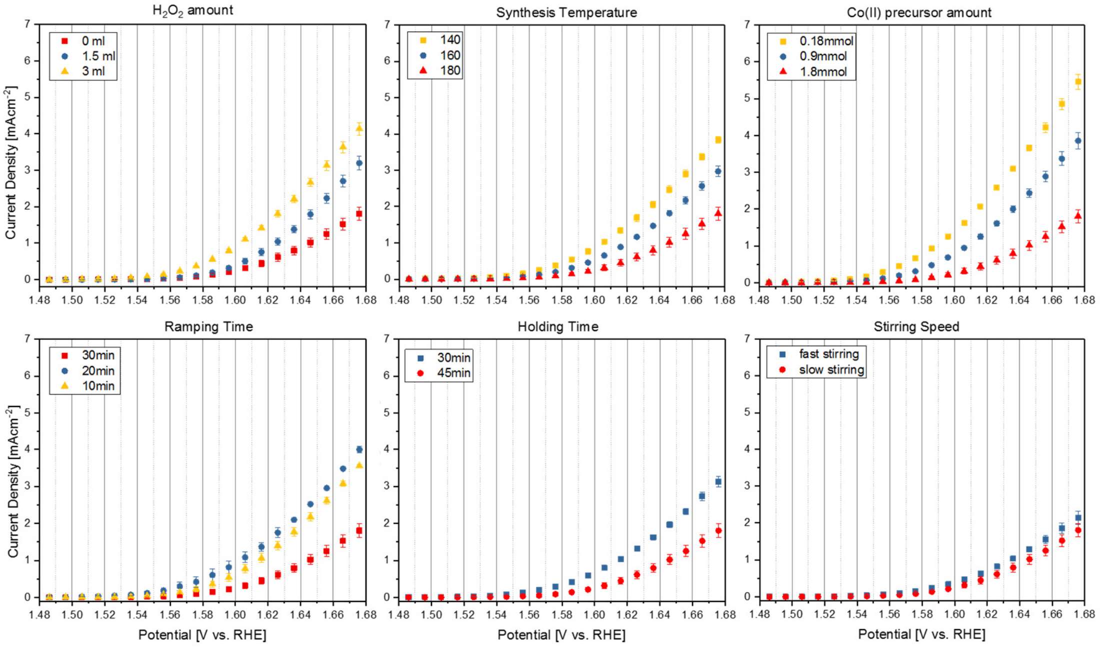

Supplementary Figure S7. Chronoamperometry measurements of all samples from 1.48 – 1.68 V vs. RHE with 0.1 V steps and a holding time of 5 min for each step. For the sake of clarity, only the stabilized current after 5 min holding is plotted against the potential.

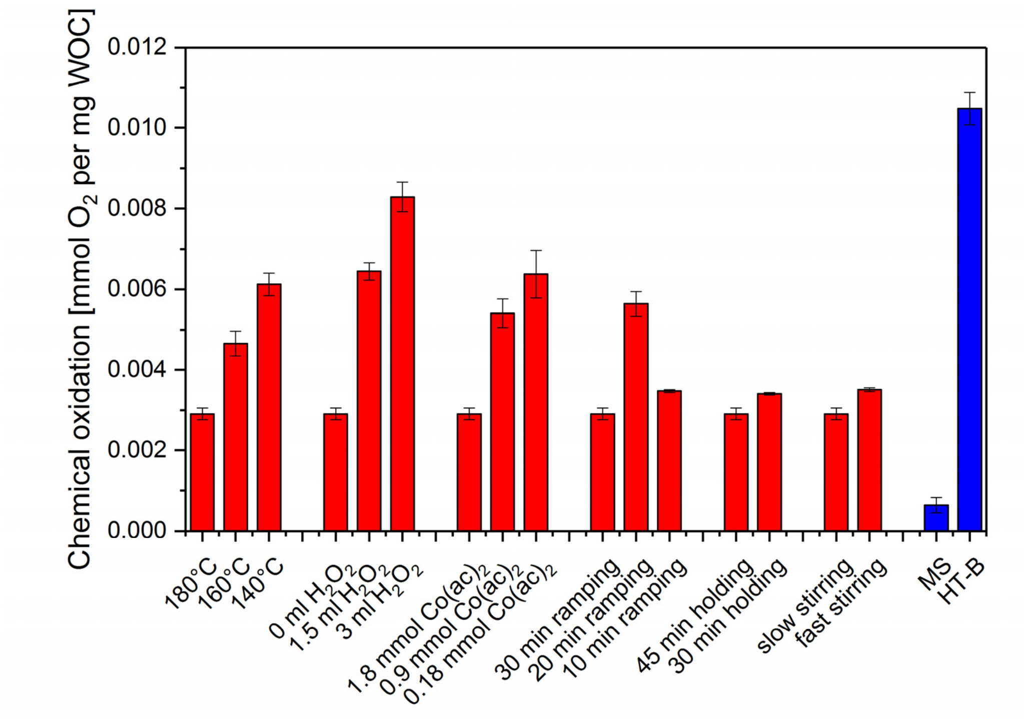

Supplementary Figure S8. Chemical water oxidation activity values recorded in this work compared to the least and most active samples from our recent comparison of Co_3O_4 synthesis methods.[1]

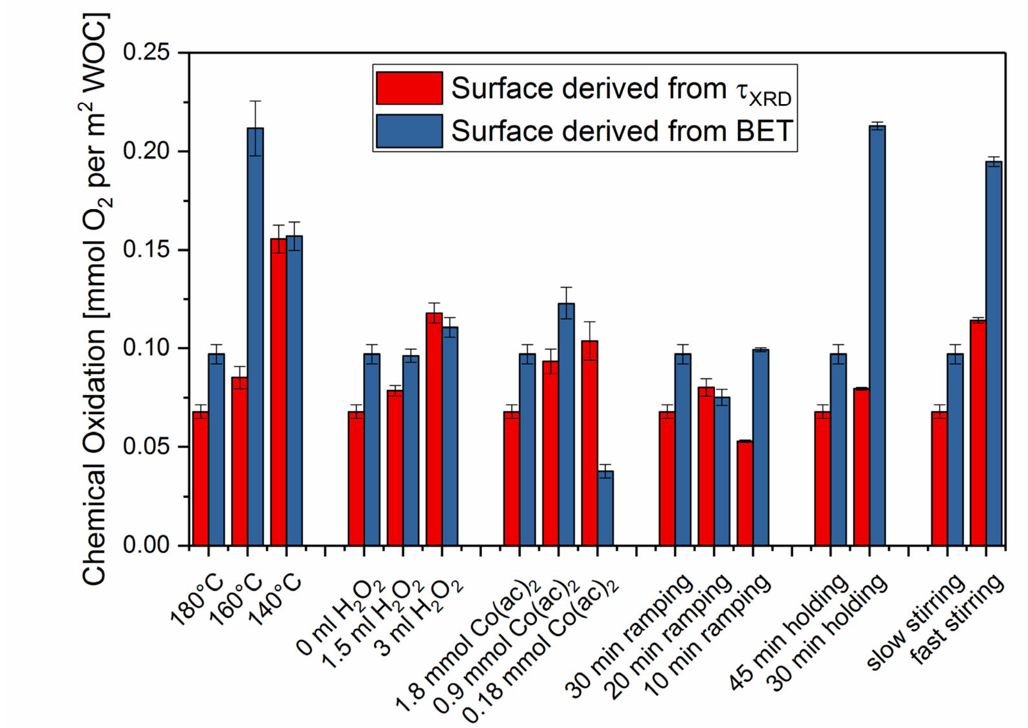

Supplementary Figure S9. Chemical water oxidation activity normalized to the WOC surface area determined with two different methods, namely (1) calculation from crystallite sizes determined by the Scherrer equation at FWHM of the PXRD patterns and (2) BET measurements.

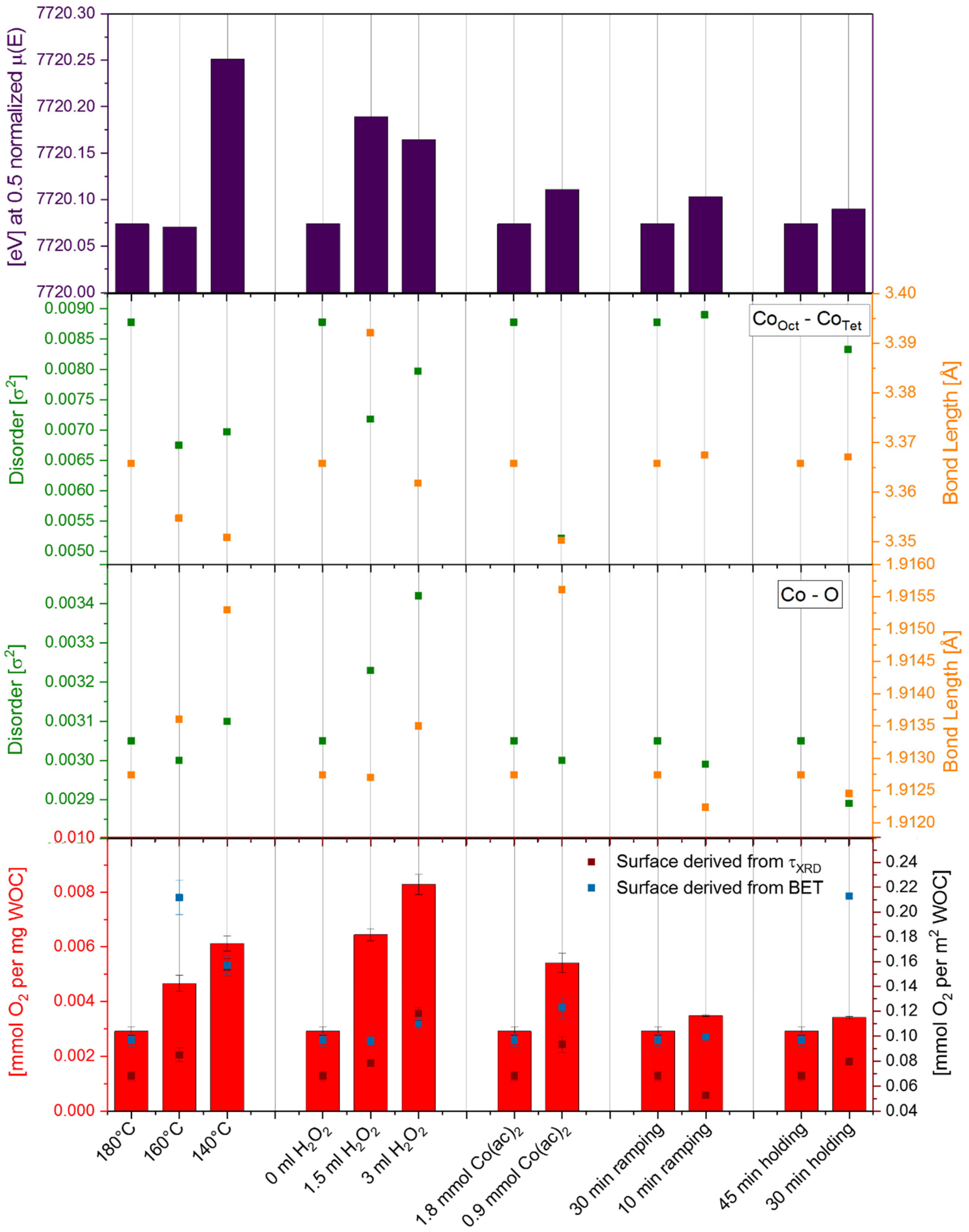

Supplementary Figure S10. Chemical water oxidation activity (normalized to surface area and not normalized) together with the disorder and bond length values of Co-O and Co_{Oct}-Co_{Tet}, respectively, and the XANES energy at 0.5 normalized μE as a measure of the overall cobalt oxidation state.

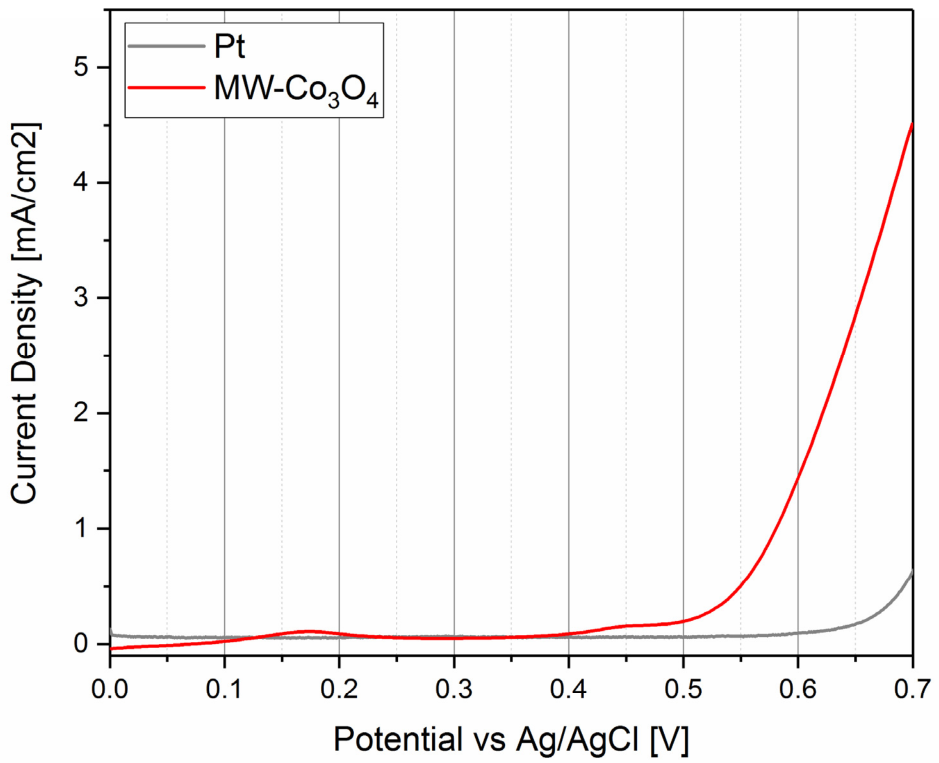

Supplementary Figure S11. LSV measurements of standard MW-Co_3O_4 in comparison with a Pt electrode in 1 M KOH.

LSV measurements of a representative standard MW-Co_3O_4 catalyst and a plain Pt working electrode reveal the very low activity of Pt in the applied potential range. This data and the reported minimized dissolution of Pt in basic electrolytes justifies the use of Pt as a counter electrode material.[2]

EIS and long-term chronoamperometry measurements:

The standard Co_3O_4 sample and the sample synthesized at 140 °C were chosen for electrochemical impedance spectroscopy (EIS) and long-term chronoamperometry measurements. The better catalytic activity of the sample prepared at 140 °C might arise from the lower resistivity towards OER on the electrode surface compared to the standard sample. To test this hypothesis, EIS measurements of the above-mentioned samples were carried out in the three-electrode configuration in 1.0 M KOH. The Nyquist plots of both electrodes show a semicircle in the frequency range of 0.1-50 kHz (Figure S12a), which can mainly be correlated to charge transfer resistance (R_{ct}) of the cobalt oxide catalysts. The diameter of the semicircle in the Nyquist diagram of Co_3O_4 synthesized at 140 °C is smaller in comparison with the standard sample, showing lower R_{ct}, due to reduced resistivity of OER on the electrode surface of the 140 °C sample. A coated electrode equivalent electrical circuit was used to fit the EIS data, and the R_{ct} of the 140 °C sample at 1.69 V (vs RHE) is estimated as 6.20 Ω cm^{-2}, i.e. lower than that of the standard sample (8.40 Ω cm^{-2}). This is presented in the Bode plots (Figure S12b) as well, where the sample synthesized at 140 °C shows lower resistance (Log |Z|). Therefore, it was shown that the standard sample suffers from high OER resistivity in the applied potential window. The lower OER resistivity of the 140 °C sample results in enhanced charge transfer properties.

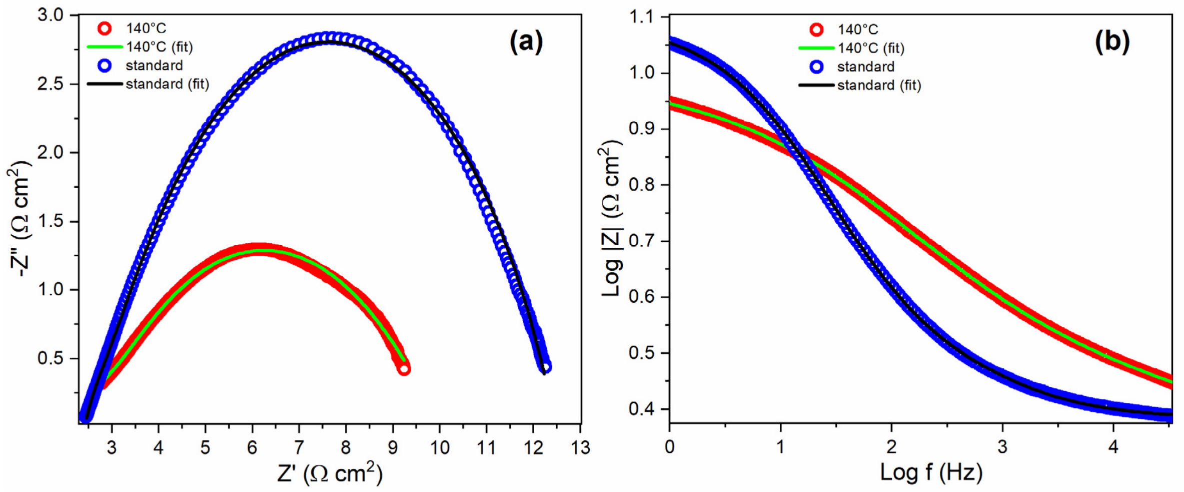

Supplementary Figure S12. (a) Nyquist and (b) Bode plots of Co_3O_4 samples (standard procedure and synthesized at 140 °C) in 1.0 M KOH at 1.69 V vs RHE applied potential in the frequency range of 0.1 Hz–50 kHz.

Supplementary Table S2. Equivalent circuit and its parameters for Carbon paper/catalyst in 1.0 M KOH solution obtained from the Nyquist plots of Figure S12.

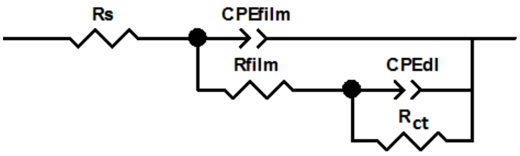

Element	Value	
	140 °C	Standard
R_S	2.03	2.41
CPE_{film}-T	8.09×10^{-3}	3.54×10^{-3}
CPE_{film}-P	0.37	0.66
R_{film}	1.77	1.66
CPE_{dl}-T	6.57×10^{-3}	3.98×10^{-3}
CPE_{dl}-P	0.48	0.67
R_{ct}	**6.20**	**8.40**

To evaluate the durability of the catalyst synthesized at a lower temperature of 140 °C for water oxidation, long-term chronoamperometry was carried out at 2.19 V vs RHE in 1.0 M KOH for more than 20 h. In comparison with the standard sample, the sample obtained at 140 °C showed higher current density in the long-term measurement. As shown in Figure S13, a higher current density of ~10.0 mA cm^{-2} was recorded for the 140 °C sample compared to the standard Co_3O_4 (~7.0 mA cm^{-2}). The activity loss with time could be explained by the surface amorphization or decreasing physical stability of the Nafion layer on the electrode surface.

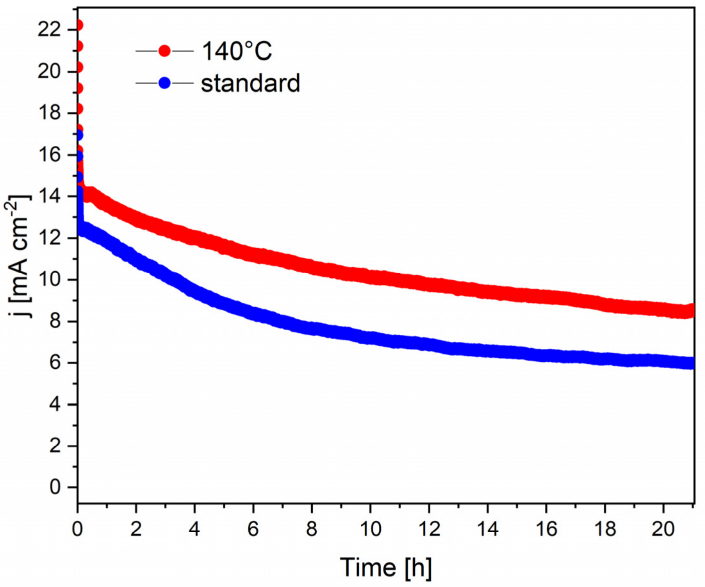

Supplementary Figure S13. Long-term chronoamperometry of Co_3O_4 samples (standard conditions and synthesized at 140 °C) in 1.0 M KOH at 1.64 V vs RHE.

III. References

[1] L. Reith, K. Lienau, C. A. Triana, S. Siol, and G. R. Patzke, Preparative History vs Driving Force in Water Oxidation Catalysis: Parameter Space Studies of Cobalt Spinels, *ACS Omega* **2019**, *4*, 15444−15456.

[2] S. Cherevko, A. R. Zeradjanin, G. P. Keeley, K. J. J Mayrhofer, A Comparative Study on Gold and Platinum Dissolution in Acidic and Alkaline Media. *J. Electrochem. Soc.* **2014**, *161*, 822-830.
